# Rab27a-mediated extracellular vesicle secretion contributes to osteogenesis in periodontal ligament-bone niche communication

**DOI:** 10.1038/s41598-023-35172-x

**Published:** 2023-05-25

**Authors:** Yun Lu, Liru Zhao, Jiaqi Mao, Wen Liu, Wensheng Ma, Bingjiao Zhao

**Affiliations:** 1 Department of Orthodontics, Shanghai Stomatological Hospital & School of Stomatology, Fudan University, Shanghai, 200001 China; 2 Shanghai Key Laboratory of Craniomaxillofacial Development and Diseases, Fudan University, Shanghai, 200001 China; 3 Department of Orthodontics, Hebei Key Laboratory of Stomatology, Hebei Clinical Research Center for Oral Diseases, School and Hospital of Stomatology, Hebei Medical University, Shijiazhuang, 050017 China

**Keywords:** Mesenchymal stem cells, Periodontitis, Regeneration

## Abstract

Periodontitis, an infectious and common disease worldwide, leads to the destruction of the periodontal ligament-alveolar bone complex. Within the bone metabolic niche, communication between periodontal ligament stem cells (PDLSCs) and bone marrow mesenchymal stem cells (BMMSCs) has been considered a major contributor to osteogenesis. PDLSC-derived extracellular vesicles (P-EVs) have shown great potential for bone regeneration. However, the secretion and uptake mechanisms of P-EVs remain elusive. Herein, the biogenesis of extracellular vesicles (EVs) from PDLSCs was observed using scanning and transmission electron microscopy. PDLSCs were transduced with Ras-associated protein 27a (Rab27a) siRNA (PDLSC^*siRab27a*^) to inhibit EV secretion. The effect of P-EVs on BMMSCs was evaluated using a non-contact transwell co-culture system. We observed that Rab27a knockdown decreased EV secretion, and PDLSC^*siRab27a*^ remarkably attenuated co-culture-enhanced osteogenesis of BMMSCs. Isolated PDLSC-derived EVs enhanced osteogenic differentiation of BMMSCs in vitro and induced bone regeneration in a calvarial defect model in vivo. PDLSC-derived EVs were rapidly endocytosed by BMMSCs via the lipid raft/cholesterol endocytosis pathway and triggered the phosphorylation of extracellular signal-regulated kinase 1/2. In conclusion, PDLSCs contribute to the osteogenesis of BMMSCs through Rab27a-mediated EV secretion, thereby providing a potential cell-free approach for bone regeneration.

## Introduction

The periodontium of a human tooth is a complex structural unit that includes the cementum, periodontal ligament (PDL), and alveolar bone. PDL, a dense connective tissue that attaches the tooth to the alveolar bone, represents a cell renewal system for periodontal bone remodeling^1–3^. A healthy PDL-bone interface is capable of processing adaptive remodeling in age-related changes in natural tooth arrangement and external tooth movement caused by orthodontic forces. During periodontitis, the biological role of the PDL-bone interface is dysregulated by the destruction of the PDL and alveolar bone. Periodontal ligament stem cells (PDLSCs), the main progenitor cells present in PDL tissue, have been identified as mesenchymal stem cells (MSCs) and are considered suitable seed cells for periodontal tissue regeneration^4,5^. Experiments in vivo and in vitro have shown that PDLSCs, with the capacity to provide paracrine factors, indirectly contribute to alveolar bone regeneration to enhance osteogenic activity of nearby cells, such as bone marrow mesenchymal stem cells (BMMSCs)^6–8^.

Alveolar bone marrow has also been established as a cell source for regenerative medicine, and alveolar bone-derived BMMSCs exhibit reinforced in situ osteogenicity compared to iliac bone-derived BMMSCs^9,10^. As two prominent stem cells exist in the periodontium, utilizing the intercellular crosstalk between PDLSCs and BMMSCs may be beneficial for bone remodeling. For example, using an indirect co-culture system, PDLSCs were shown to enhance BMMSC osteogenicity, whereas gingival fibroblasts did not^7,8^. PDLSC-derived conditioned medium reportedly promoted M2 macrophage polarization and enhanced alveolar bone regeneration, suggesting that PDLSC-derived paracrine factors could contribute to bone regeneration^11,12^.

An increasing and convincing body of evidence shows that MSCs exert paracrine effects primarily by releasing extracellular vesicles (EVs) that are 50–1000 nm in diameter^13,14^. Small EVs (sEVs, 50–200 nm in diameter) reportedly have potential therapeutic effects in various preclinical models^13,15,16^. PDLSC-derived EVs (P-EVs) may also play a crucial role in periodontal cellular communication and periodontal tissue homeostasis, although their secretion and uptake mechanisms remain elusive^11,17–19^.

The biological role of EVs depends on the origin and recipient cells^20^. P-EVs enhance BMMSC migration and induce bone repair via the adenosine receptor signaling pathway^21^. Mechanical force alters the protein cargo in P-EVs, which facilitates EV internalization and activates the phosphorylation of extracellular signal-regulated kinase (ERK)^19^. In this study, we aimed to examine whether PDLSCs participate in osteogenic differentiation of BMMSCs through the release of EVs. To that end, we developed PDLSC^*siRab27a*^ in which Ras-associated protein 27a (Rab27a) was knocked down using siRNA. As a driver of sEV release, Rab27a controls the fusion of multivesicular endosomes (MVEs) with the plasma membrane, initiating sEV release into the extracellular space^22^. Rab27a downregulation in PDLSCs inhibited EV production and abrogated the co-culture-enhanced osteogenic activity of BMMSCs. EVs isolated from PDLSC culture medium enhanced BMMSC osteogenesis in vitro and in vivo. Additionally, BMMSC internalization of P-EVs was shown to be mediated by the lipid raft/cholesterol endocytosis pathway. These findings provide valuable insights into the mechanisms of secretion and uptake in cellular communication during periodontal tissue remodeling.

## Materials and methods

### Cell culture

Extracted premolars from three healthy donors aged 11–13 years were collected with informed consent from their guardians. All collected teeth had intact roots without caries or periodontitis. This study was approved by the Experiment and Ethics Committees of Shanghai Stomatological Hospital, Fudan University (NO. 20170007). PDL tissues were gently scraped from the middle third of the root, minced, and incubated for 10 d. Limiting dilution technique was used to isolate PDLSCs^5^. Briefly, single-cell suspensions of primary PDL cells (1 × 10^3^ cells/mL) were seeded into 96-well plates with complete culture medium containing αMEM (Gibco, Gaithersburg, MD, USA), 10% fetal bovine serum (Gibco), 1% antibiotic–antimycotic solution (Gibco), and 1% L-glutamine (Invitrogen, Carlsbad, CA, USA). On day 10 of culture, aggregates of 50 or more were scored as colonies and trypsinized for sub-culture. PDLSCs from passages 4–5 were used for the following studies.

Human BMMSCs were obtained from the National Collection of Authenticated Cell Cultures (SCSP-405; ATCC number: PCS-500-012™). Cells were maintained in growth medium containing NutriStem® MSC XF Basal Medium (05–200-1A, Biological Industries, Kibbutz Beit-Haemek, Israel) supplemented with 0.6% NutriStem® MSC XF Supplement (05-200-1U, Biological Industries) and 1% antibiotic–antimycotic solution (Gibco). Osteogenic medium, composed of growth medium, 50 μg/mL ascorbic acid (Sigma-Aldrich, St. Louis, MO, USA), 100 nM dexamethasone (Sigma-Aldrich), and 10 mM β-glycerophosphate (Sigma-Aldrich), was used for osteogenesis.

### Flow cytometry analysis

The expression of typical surface markers in PDLSCs at the third passage was analyzed using a Novocyte Flow Cytometer (ACEA Biosciences, San Diego, CA, USA). The adherent cells were collected and resuspended in 50 μL of phosphate-buffered saline (PBS). The harvested cells were stained with 5 μL antibodies, namely FITC anti-human CD31 (#303103, BioLegend, San Diego, CA, USA), FITC anti-human CD34 (#343603, BioLegend), FITC anti-human CD73 (#344016, BioLegend), FITC anti-human CD90 (#328107, BioLegend), or FITC anti-human CD146 (#361011, BioLegend), in the dark for 30 min at 4 °C. Raw data were analyzed using the FlowJo software (FlowJo, Ashland, OR, USA).

### Transmission electron microscopy (TEM)

EV secretion by PDLSCs was observed using TEM. The cells were collected by centrifugation and fixed overnight in 2.5% glutaraldehyde, followed by fixation with 1% osmium tetraoxide. The samples were then dehydrated in gradient concentrations of ethanol and embedded in an epoxy resin. Ultrathin sections (approximately 70 nm) were stained with 3% uranyl acetate and lead citrate for observation.

To observe the morphology of P-EVs, 20 µL of the collected EV suspension was poured on formvar-carbon-coated copper grids and incubated with uranyl acetate. After removing excess stain, images were recorded using a Tecnai G2 Spirit transmission electron microscope (FEI Company, Brno, Czech Republic) at 100 kV.

### Scanning electron microscopy (SEM)

Coverslips were placed in a 12-well plate, on which PDLSCs were cultured at a density of 5 × 10^3^ cells/well overnight. After culturing in serum-free medium for 24 h, PDLSCs were immersed in the fixative for 24 h at 4 °C and dehydrated in an acetone dilution series. After critical point drying, the samples were gold-coated and observed using a scanning electron microscope (SU8000, Hitachi, Japan). The vesicle diameter was measured using the ImageJ2 × software (National Institutes of Health, Bethesda, MD, USA).

### Rab27a knockdown in PDLSCs

To develop PDLSC^*siRab27a*^, PDLSCs were placed in 6-well plates at a density of 2 × 10^5^ cells/well and incubated until 80% confluence. The cells were then transfected with Rab27a siRNA (h) (sc-41834, Santa Cruz Biotechnology, Inc., Dallas, TX, USA) or control siRNA (sc-37007, Santa Cruz Biotechnology). For each well, 7.5 μL of siRNA duplex was diluted with 125 μL Opti-MEM™ (Invitrogen) as Solution A, and 7.5 μL of Lipofectamine™ 3000 reagent (Invitrogen) was diluted with 125 μL Opti-MEM™ as Solution B. Solutions A and B were gently mixed and incubated for 15 min at room temperature. The siRNA mixture was then added to the PDLSCs, which were incubated for 48 h. The efficiency of siRNA-mediated transfection of PDLSC^*siRab27a*^ was evaluated using reverse transcription polymerase chain reaction (RT-PCR) and western blotting analysis relative to the control cells. The primer sequences used are listed in Table 1. The primary antibody used for western blotting was mouse anti-Rab27a (E-8) (sc74586, Santa Cruz).

**Table 1:** Primer sequences for quantitative RT-PCR.

Gene name	Primer sequences	
	Forward	Reverse
*Rab27a*	GGAGAGGTTTCGTAGCTTAACG	CCACACAGCACTATATCTGGGT
*COL1*	GAGGGCCAAGACGAAGACATC	CAGATCACGTCATCGCACAAC
*ALP*	AACATCAGGGACATTGACGTG	GTATCTCGGTTTGAAGCTCTTCC
*RUNX2*	TGGTTACTGTCATGGCGGGTA	TCTCAGATCGTTGAACCTTGCTA
*BSP*	CACTGGAGCCAATGCAGAAGA	TGGTGGGGTTGTAGGTTCAAA
*β-actin*	CATGTACGTTGCTATCCAGGC	CTCCTTAATGTCACGCACGAT

*RT-PCR* reverse transcription polymerase chain reaction.

### Gene expression assays

Total RNA was extracted using the TRIzol reagent (Invitrogen). cDNA was produced from total RNA using a cDNA Synthesis Kit (R7026, TIANGEN, Beijing, China). RT-PCR was performed using the SYBR Green method (U8190, TIANGEN), consisting of 40 amplification cycles (denaturation at 95 ℃ for 10 s, annealing at 60 ℃ for 30 s, and elongation at 70 ℃ for 20 s). Osteogenic gene expression levels for collagen type I (*COL1*), alkaline phosphatase (*ALP*), runt-related transcription factor 2 (*RUNX2*), and bone sialoprotein (*BSP*) were calculated by normalizing the values to those of the endogenous control β-actin. The primer sequences used are listed in Table 1.

### Western blotting analysis

Cells were added to 2 × lysis buffer (P0013K, Beyotime, Shanghai, China) containing a cocktail of protease inhibitors. Protein concentration was quantified using the Pierce bicinchoninic acid (BCA) Protein Assay Kit (23227, Thermo Scientific). Proteins were separated using a 10% sodium dodecyl sulfate–polyacrylamide gel and then transferred onto a polyvinylidene fluoride membrane. The primary antibodies used were rabbit anti-RUNX2 (dilution 1:1000, #343581, AbMART), anti-ALP (dilution 1:1000, ab95462, Abcam), anti-ERK1/2 (dilution 1:1000, #4695, Cell Signaling Technology), anti-phospho-ERK1/2 (dilution 1:1000, #4370, Cell Signaling Technology), and mouse anti-GAPDH (dilution 1:2000, sc32233; Santa Cruz). Protein blots were visualized using an Amersham Imager 600 (GE HealthCare, Chicago, IL, USA), and the images were analyzed using the ImageJ software. Relative protein levels were normalized to GAPDH, which served as the control.

### Isolation and identification of EVs

The PDLSC supernatant was collected after 48 h of incubation in a serum-free medium. EVs were isolated by ultracentrifugation according to previously described standard methods^16^. The medium was centrifuged at 2000 × *g* for 30 min, followed by centrifugation at 10,000 × *g* for 15 min. The collected supernatant was filtered using a 0.22-μm pore size filter, followed by ultracentrifugation at 100,000 × *g* for 70 min. Optima XPN-100 (Beckman Coulter, Brea, CA, USA) was used to perform ultracentrifugation.

EV secretion from PDLSC^*siControl*^ and PDLSC^*siRab27a*^ cells was evaluated by size distribution, particle concentration, and protein concentration. EVs were lysed in pre-cold radio-immunoprecipitation buffer, and protein concentration was measured using the Pierce™ BCA protein assay kit (Thermo Fisher Scientific, Rockford, IL, USA). To measure particle concentration and size distribution, a nanoscale flow cytometer (N30E, NanoFCM, XiaMen, China) was calibrated using 250-nm polystyrene beads with a defined concentration of 2.01 × 10^10^. Silica nanosphere cocktails were used as size reference standards and PBS served as the background signal. EVs were diluted 100-fold in PBS, and data were collected for 60 s at a sample pressure of 1.0 kPa to produce histograms of each distribution. NanoFCM software (NF Profession V2.0, NanoFCM Co. Ltd.) was used to record the raw data.

Expression levels of EV markers, including CD63 (dilution 1:300, sc5275, Santa Cruz), CD9 (dilution 1:500, sc13118), CD81 (dilution 1:300, sc23962), and TSG101 (dilution 1:300, sc7964), were evaluated using western blotting. Briefly, PDLSCs and P-EVs were treated with a pre-cold lysis buffer. Proteins were then extracted and quantified by the Micro BCA protein assay (23235, Thermo Scientific) according to the manufacturer’s instructions. Western blotting analysis was performed as previously described procedures in *2.7*.

### Transwell assay

A non-contact co-culture system was established to investigate cellular crosstalk between PDLSC^*siRab27a*^/PDLSC^*siControl*^ and BMMSCs. BMMSCs were seeded at a density of 2 × 10^4^ cells/well in the bottom, and PDLSC^*siRab27a*^/PDLSC^*siControl*^ were seeded at a density of 1 × 10^5^ cells/well in the top well inserts (pore size 0.4 mm; #3412, Corning, Kennebunk, USA) of a Transwell plate. In this system, only the paracrine factors of the upper cells affected BMMSC performance. After 3 d of osteogenic induction, total RNA was extracted from cultured BMMSCs, and mRNA expression was evaluated using RT-PCR. After 3 weeks of osteogenic induction, mineral deposition of the cultures was stained with an Alizarin Red-S (ARS) staining kit (GMS80046.3, Genmed, Arlington, TX, USA). BMMSCs were fixed with 4% paraformaldehyde, washed twice, and stained. Photographs were taken after washing five times with distilled water. For semi-quantitative analysis, the deposition was dissolved in 10% cetylpyridinium chloride (Sigma-Aldrich) and examined via a colorimetric assay at an absorbance of 562 nm.

### Determination of P-EV contribution in BMMSC osteogenic differentiation in vitro

BMMSCs (5 × 10^4^) were seeded in 24-well plates and cultured in an osteogenic medium for osteogenic induction. To identify the direct role of P-EVs in cell communication, BMMSCs were treated with P-EVs (1, 5, or 10 µg/mL) or an equivalent volume of PBS as a control. The medium was changed twice per week. After 7 d, ALP staining was performed using the 5-bromo-4-chloro-3-indolyl phosphate (BCIP)/nitro blue tetrazolium (NBT) ALP color development kit (C3206, Beyotime, Shanghai, China); cells were fixed with 4% paraformaldehyde, washed twice with PBS, and stained with the BCIP/NBT working solution. Staining was visualized using a stereoscope (M80, Leica Microsystems Inc, Wetzlar, Germany) and observed under a bright-field microscope. To measure ALP activity, cells were washed twice and lysed with 60 μL of lysis buffer (P0013F, Beyotime). The cell lysates were semi-quantified using an ALP assay kit (P0321S, Beyotime) with p-nitrophenyl phosphate as the substrate. ALP activity was determined using colorimetry at an absorbance of 405 nm. After 2 weeks, P-EV-treated BMMSCs and control cells were fixed for ARS staining and quantification to evaluate mineral deposition, as previously described.

### Endocytosis assay

To verify P-EV entry into BMMSCs, EVs were fluorescently labeled using the PKH26 Red Fluorescent Cell Linker Kit (MIDI26, Sigma-Aldrich, Australia), according to the manufacturer’s protocol. Briefly, 100 µg of P-EVs was mixed with 250 μL of Diluent C (Part A), and the staining solution was prepared by adding 1 μL of PKH26 dye solution to 250 μL of Diluent C (Part B). After incubating Part A with Part B for 5 min, the reaction was stopped using 500 μL of 1% bovine serum albumin. The mixture was washed with PBS, and EVs were isolated by ultracentrifugation at 100,000 × *g* for 70 min. For fluorescence observation of EV entry into BMMSCs over a period of 24 h, 2 × 10^4^ BMMSCs were seeded on glass coverslips. PKH26-labeled EVs (10 μg/mL) were added and incubated for 24 h at 37 ℃. After washing with PBS, BMMSCs were fixed with 4% paraformaldehyde. The nuclear stain 4′,6-diamidino-2-phenylindole and ProLong Diamond Antifade Mountant (Thermo Fisher Scientific) were added to the coverslips. Representative images of the slides were captured using a fluorescence microscope (Leica Microsystems Inc.).

Blocking experiments were performed to assess whether P-EV uptake was mediated by dynamin or associated with cholesterol-rich membrane microdomains. BMMSCs were pretreated with dynasore [100 μM from a stock solution of 100 mM in dimethyl sulfoxide (DMSO); HY-15304, MCE], methyl-β-cyclodextrin (MβCD, 250 μM from a stock solution of 100 mM in DMSO) (ST1515, Beyotime) for 1 h, or cultured with simvastatin (5 μM from stock solution of 1 mM in dH_2_O; 567,022, Millipore) for 6 h. Control cells were treated with the same concentration of DMSO or dH_2_O. PKH-26-labeled P-EVs were added to the cells and incubated for 4 h. EV internalization was captured using a fluorescence microscope and assessed by fluorescence quantitation using the ImageJ software in five randomly selected fields.

### In vivo experiments in a rat calvarial defect model

Male Sprague Dawley rats (age: 8 weeks, weight: 200–250 g), used for the in vivo experiments, were housed in a specific-pathogen-free environment with an artificial 12/12 h light/dark cycle. All rats were anesthetized via intraperitoneal injection of 2% pentobarbital sodium (0.3 mL/100 g body weight), followed by bilateral calvarial defect surgery. Two 5-mm diameter full-thickness defects were created in the calvarium using a trephine bur with simultaneous saline irrigation to avoid thermal-induced osteonecrosis. The two calvarial defects were randomly assigned to either the control or EV groups (n = 10 each). For the EV group, 20 μg P-EVs (in 10 μL PBS) were mixed with 10 μL Matrigel and pressed to carefully fit into the defect. Mixed PBS and Matrigel were used as the controls. Calvarial defect healing was evaluated after 8 weeks. A histological evaluation, which included hematoxylin and eosin (H&E) and Masson’s trichrome staining, was then performed. For Masson’s staining analysis, the collagen matrix area was calculated as the percentage of blue-stained tissue area per total area at five representative microphotographs. Bone regeneration was evaluated using immunohistochemical (IHC) staining for ALP and osteopontin (OPN). For quantification of ALP and OPN, positively stained cells were counted and expressed as a percentage of positively stained cells.

### Statistical analysis

All quantitative data are expressed as the mean ± standard deviation (SD). Shapiro–Wilk test was used to evaluate the normality of the data distribution. Mean differences between groups were analysed using one-way ANOVA followed by a post-hoc SNK test for normally distributed data. Non-parametric data was analysed using the Mann–Whitney U test. For siRNA assays, statistical analysis between siRab27a and siControl was performed using pairwise one-tailed Student’s* t*-tests. Statistical analysis was performed using the SPSS software, version 20.0 (SPSS Inc., USA). Results were considered statistically significant at *p* < 0.05.

### Institutional review board statement

The study was conducted according to the guidelines of the Declaration of Helsinki, and approved by the Ethics Committee of the Shanghai Stomatological Hospital (approval no. [2017] 0007).

### Informed consent

Written informed consent was obtained from all donors who underwent premolar molar extraction for orthodontic treatment in Shanghai Stomatological Hospital.

## Results

### Characterization and EV biogenesis of PDLSCs

**Figure 1:**
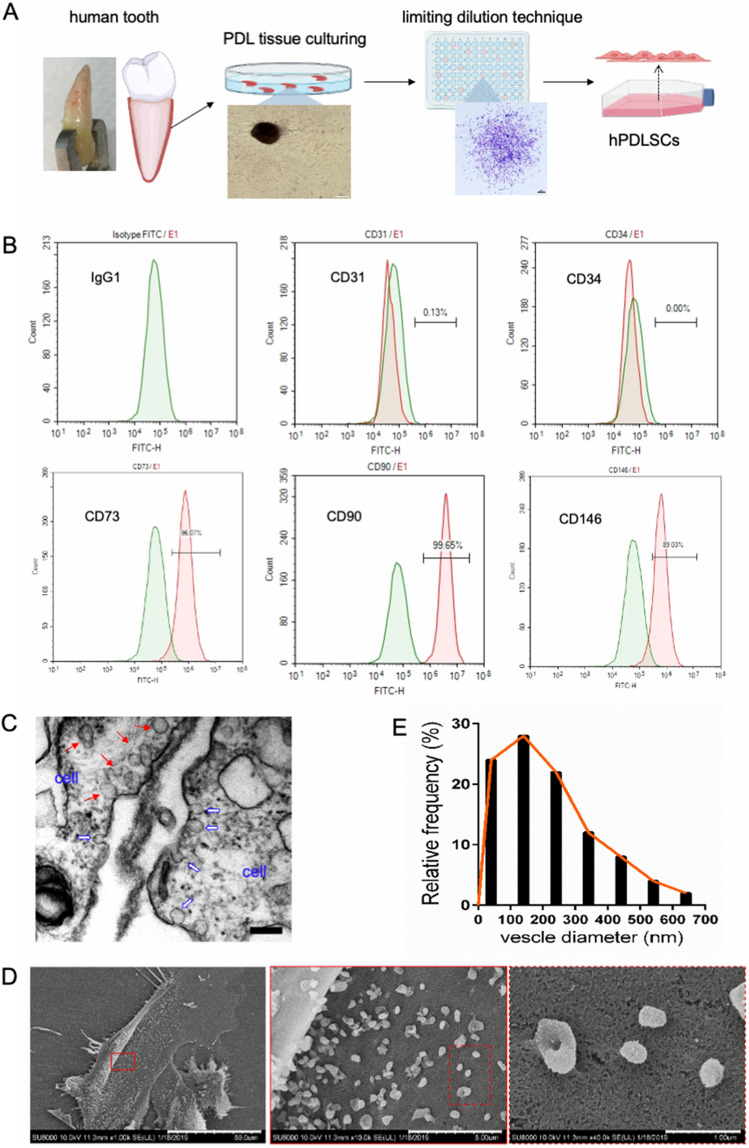
Observation of extracellular vesicle (EV) secretion by periodontal ligament stem cells (PDLSCs). (**A**) Schematic plot of PDLSC isolation from an extracted tooth. (**B**) Expression of PDLSC surface markers (CD31, CD34, CD73, CD90, and CD146) as detected using flow cytometry. (**C**) Transmission electron microscopy (TEM) image of PDLSCs showing the multivesicular body (red arrows) and inward invagination of the plasma membrane (blue arrows). Scale bars = 200 nm. (**D**) Scanning electron microscopy (SEM) images of PDLSCs, showing the presence of EVs on the cell surface. Scale bar = 50 µm (left), 5 µm (middle), and 1 µm (right). (**E**) Vesicle size distribution based on SEM images (“Supplementary information”).

Figure 1A shows a schematic diagram of PDLSCs isolated from human PDL tissues. The cultured PDLSCs were long, spindle-shaped, and arranged in a whirlpool under an inverted microscope. Flow cytometry analysis of the surface markers (Fig. 1B) showed that the positive rates of CD90, CD73, and CD146 were 99.65, 96.07, and 89.03%, respectively, while the rates of the MSC-negative markers CD31 and CD34 (expressed in endothelial cells and hematopoietic stem cells) were less than 1%. These characteristics of the cultured cells confirmed the representative features of PDLSCs. As EVs are major candidates for mediating paracrine effects, PDLSCs subjected to serum starvation were observed using TEM and SEM. TEM images showed invaginations of the plasmalemma and multivesicular bodies (Fig. 1C). As shown in Fig. 1D, PDLSCs were spread out, and many vesicles were present on the cell surface. At a higher magnification, the vesicles showed a spherical or cup-shaped morphology with diameters ranging from 40 to 640 nm. Size distribution analysis based on SEM images showed that 52% of P-EVs fell within the typical size range (40–200 nm) of sEVs and 86% of P-EVs were less than 400 nm in diameter (Fig. 1E).

### Rab27a knockdown decreases EV secretion of PDLSCs

**Figure 2:**
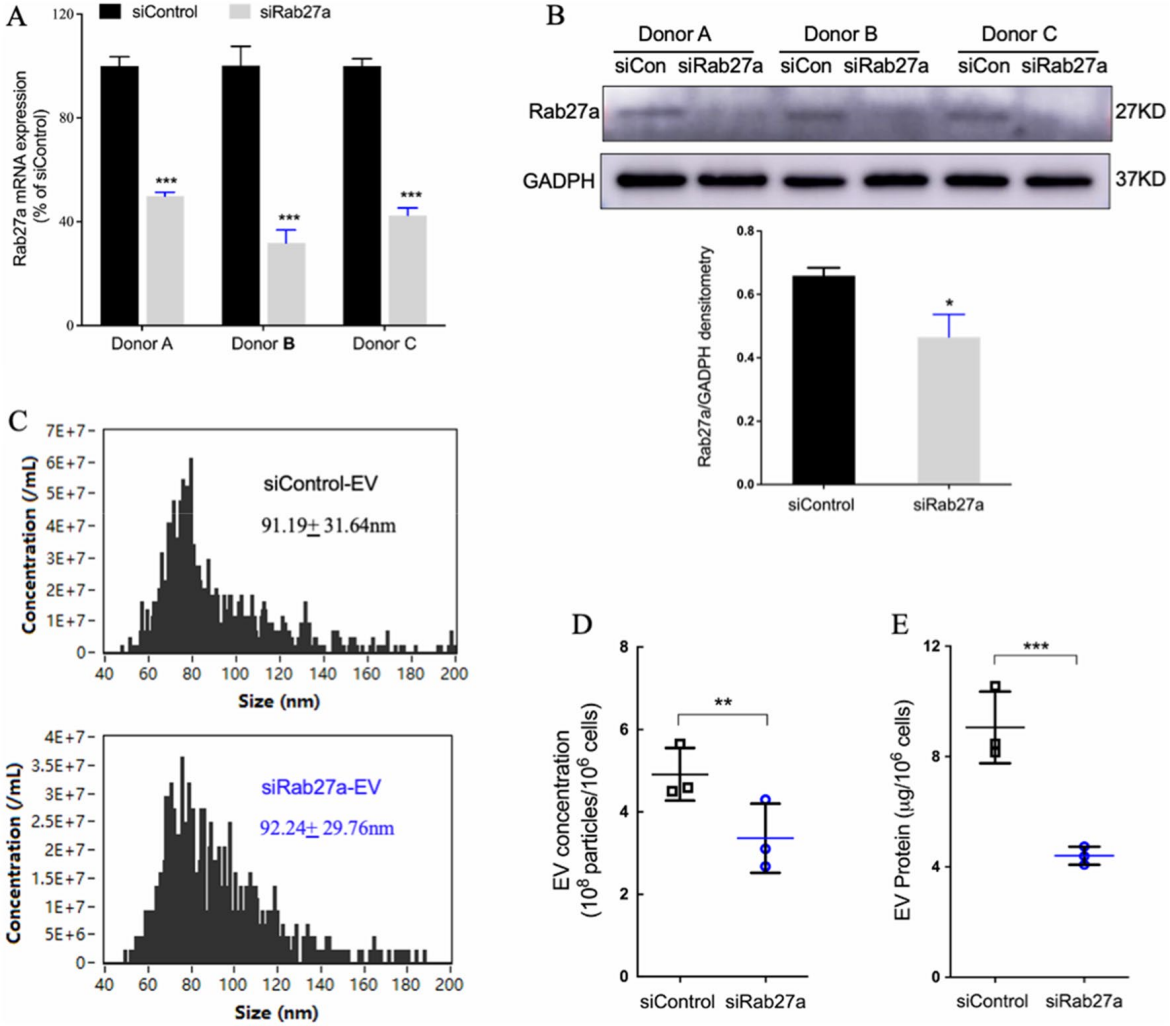
Rab27a knockdown decreases EV secretion by PDLSCs. (**A**) Relative Rab27a mRNA level in Rab27a knockdown PDLSCs (PDLSC^*siRab27a*^), compared with that in PDLSC^*siControl*^. (**B**) Rab27a expression in PDLSC^*siRab27a*^ and PDLSC^*siControl*^. *p < 0.05 as compared with the control group. (**C**) Representative nanoparticle tracking analysis (NTA) traces of EVs derived from PDLSC^*siControl*^ and PDLSC^*siRab27a*^ cells. (**D**) Quantification of EV secretion by NTA in triplicate. EVs were isolated using Exo-QC from culture supernatants of 2 × 10^6^ cells and resuspended in 10 μL of buffer. Raw data are shown as means ± standard deviation. **p < 0.01, as determined using a paired* t* test. (**E**) Protein content of EVs as calculated using bicinchoninic acid assay. ***p < 0.001, as determined using a paired* t* test.

To evaluate the role of EV secretion in PDLSC paracrine effects, siRNA was used to downregulate Rab27a expression in PDLSCs (PDLSC^*siRab27a*^). Compared to that in the control PDLSCs (PDLSC^*siControl*^), quantification via qPCR revealed more than a 50% reduction in Rab27a expression in PDLSC^*siRab27a*^ at the mRNA level (Fig. 2A). Furthermore, western blotting analysis showed an approximately 30% reduction at the protein level (Fig. 2B). To evaluate whether Rab27a knockdown could inhibit EV secretion, nanoparticle tracking analysis (NTA) and BCA protein assay were performed. The size distribution of vesicles secreted by either PDLSC^*siRab27a*^ or PDLSC^*siControl*^ was mostly ~ 95 nm (Fig. 2C). The particle concentration of PDLSC^*siRab27a*^-secreted vesicles was significantly lower than that secreted by PDLSC^*siControl*^ (Fig. 2D). The protein content of PDLSC^*siRab27a*^-secreted vesicles was less than 50% of that secreted by PDLSC^*siControl*^ (Fig. 2E).

### PDLSCs enhance BMMSC osteogenic differentiation via EV secretion

**Figure 3:**
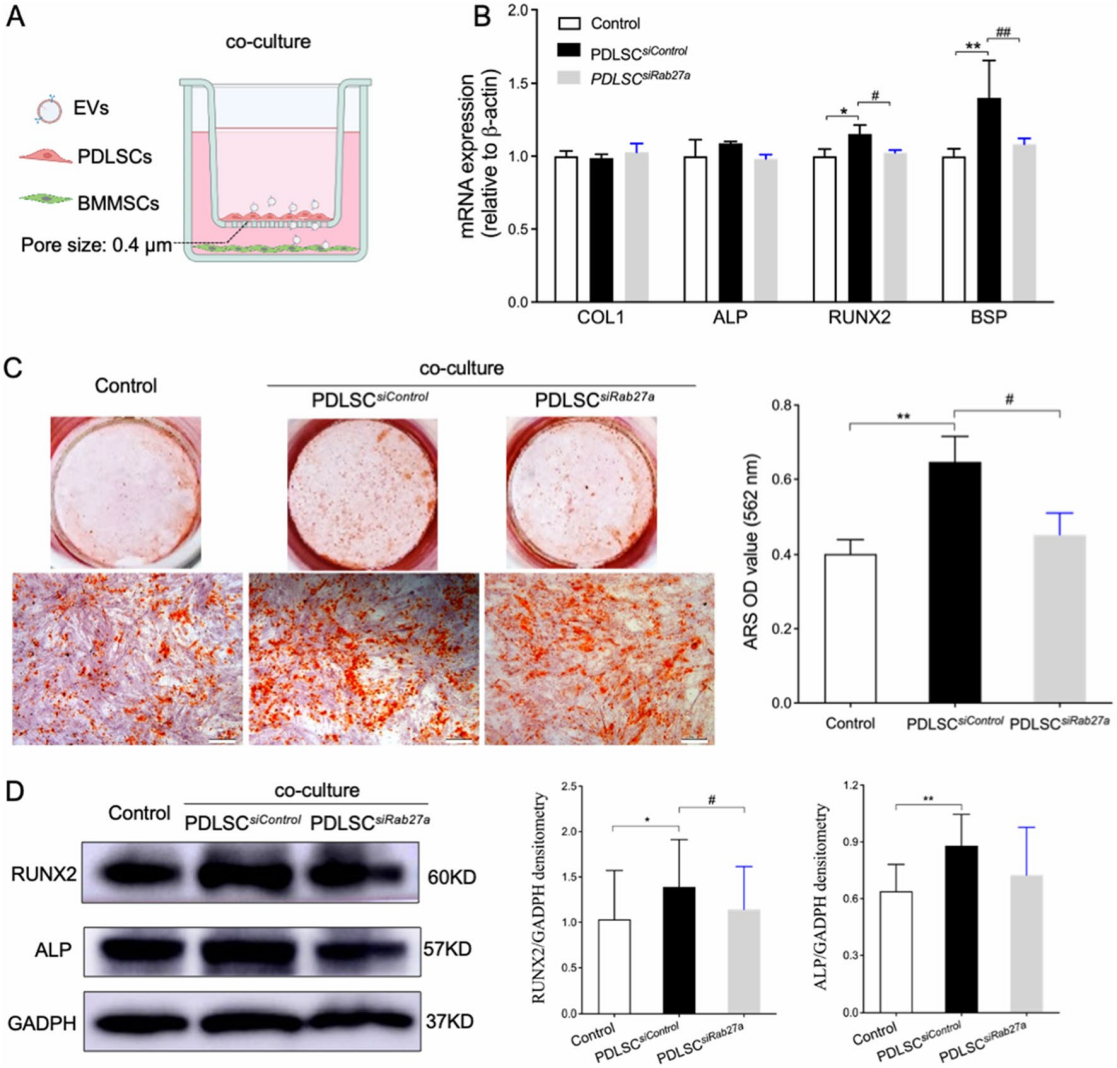
Rab27a knockdown in PDLSC-attenuated co-culture-enhanced bone marrow mesenchymal stromal cell (BMMSC) osteogenesis. (**A**) Schematic diagram of a non-contact co-culture system in which BMMSCs were cultured separately with PDLSC^*siControl*^ or PDLSC^*siRab27a*^ through a porous membrane (pore size 0.4 µm). (**B**) Osteogenic gene expression of BMMSCs in the Transwell system after 3 d of co-culture. *p < 0.05, **p < 0.01, as compared with the control group; #*p* < 0.05, ##*p* < 0.01, as compared with the PDLSC^*siControl*^ co-culture group (n = 3). (**C**) Representative Alizarin Red-S (ARS)-stained images of BMMSCs in the Transwell system after 3 weeks of co-culture. Scale bar = 200 µm. ARS intensity was evaluated using densitometry at 562 nm. **p < 0.01, as compared with the control group; #*p* < 0.05, as compared with the PDLSC^*siControl*^ co-culture group (n = 3). (**D**) Western blotting analysis revealed that co-culture with PDLSC^*siControl*^ enhanced RUNX2 expression by 1.41-fold and ALP expression by 1.38-fold. Rab27a knockdown in PDLSCs attenuated the co-culture-enhanced osteogenesis of BMMSCs. *p < 0.05 and **p < 0.01, as compared to the control group; #p < 0.05, as compared to the PDLSC^*siControl*^ co-culture group. Representative results from triplicate experiments are expressed as mean ± standard deviation.

An indirect co-culture system, in which most of P-EVs could pass through the 0.4-μm Transwell filter, was used to evaluate PDLSC paracrine effects in BMMSCs in vitro (Fig. 3A). To confirm the effect of P-EVs on BMMSC osteogenesis, PDLSC^*siControl*^ or PDLSC^*siRab27a*^ were seeded in the upper well. RT-PCR was used to assess the expression of osteogenic markers *COL1*, *ALP*, *RUNX2*, and *BSP* on day 3 (Fig. 3B). Compared to those in the control, BMMSCs in the PDLSC co-culture group showed significantly upregulated RUNX2 and BSP expression, which was attenuated in the PDLSC^*siRab27a*^ co-culture group. On day 21, ARS staining showed that PDLSC co-culture induced significantly more mineralized nodule formation in BMMSCs, whereas co-culture with PDLSC^*siRab27a*^ diminished this upregulated mineralization (Fig. 3C). Cellular osteogenesis regulation in BMMSCs was verified using western blotting for RUNX2 and ALP (Fig. 3D). These results confirmed that EV secretion by PDLSCs is beneficial for BMMSC osteogenic differentiation.

### P-EVs mediated osteogenic differentiation of BMMSCs in vitro

**Figure 4:**
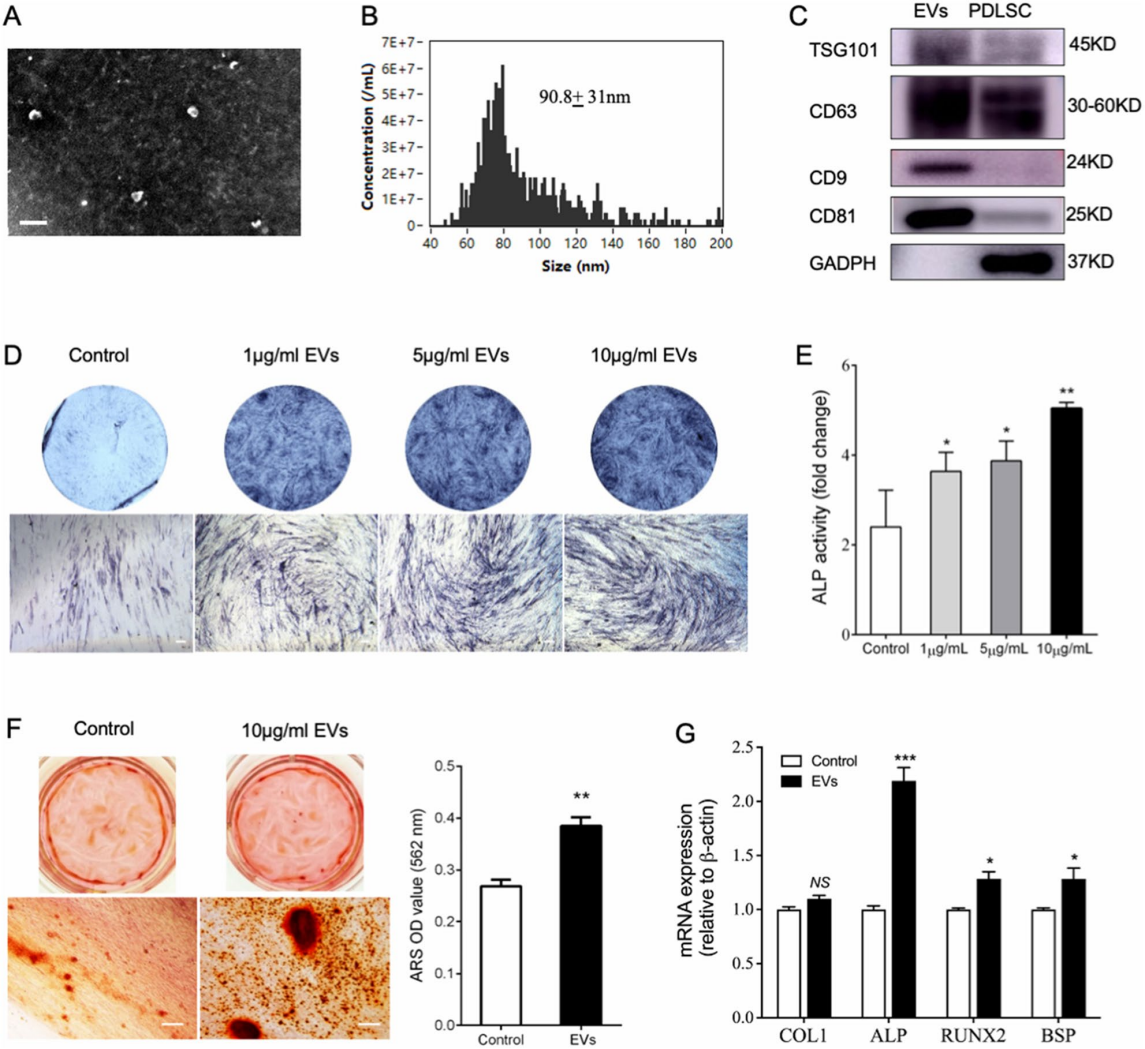
Direct effect of PDLSC-derived EVs (P-EVs) on osteogenic differentiation of BMMSCs. (**A**) Observation of P-EVs using TEM. Scale bar = 200 nm. (**B**) Particle size distribution of EVs measured using NTA; the mean size was 90.8 nm ± 31.1 (standard deviation). (**C**) Expression of EV surface markers detected using western blotting analysis. (**D**) Alkaline phosphatase (ALP) staining of BMMSCs treated with P-EVs for 7 d. (**E**) ALP activity of BMMSCs treated with P-EVs for 7 d. *p < 0.05 and **p < 0.01, as compared with the control group (n = 3). (**F**) ARS staining of BMMSCs treated with P-EVs for 14 d. ARS stain intensity was evaluated using densitometry at 562 nm. **p < 0.01, as compared with the control group (n = 3). (**G**) Osteogenic gene expression of BMMSCs with P-EV treatment on day 2. *p < 0.05 and ***p < 0.001, as compared with the control group (n = 3) (“Supplementary information”).

To identify the direct role of P-EVs in cell communication, we isolated and identified them based on their shape, size, and presence of marker proteins. TEM revealed spherical vesicles with a size of 100–150 nm (Fig. 4A). NTA showed that the size distribution of the isolated vesicles was 178.4 ± 68.8 nm (Fig. 4B). Western blotting provided evidence of the exosomal marker proteins TSG101, CD63, CD9, and CD81 in P-EVs (Fig. 4C). As a biochemical marker of osteogenesis and osteocalcin content, ALP expression in BMMSCs was measured to evaluate the effect of P-EVs on osteogenic differentiation. After 7 d, incubation of BMMSCs with P-EVs resulted in the upregulation of ALP staining and ALP activity in a dose-dependent manner (Fig. 4D, E). Moreover, ARS staining showed that P-EV treatment resulted in a significantly greater accumulation of mineralized matrix (Fig. 4F). Gene expression analysis showed that P-EVs increased the expression levels of ALP, BSP, and RUNX2 in BMMSCs treated with P-EVs for 48 h (Fig. 4G).

### P-EVs contribute to bone repair in vivo

**Figure 5:**
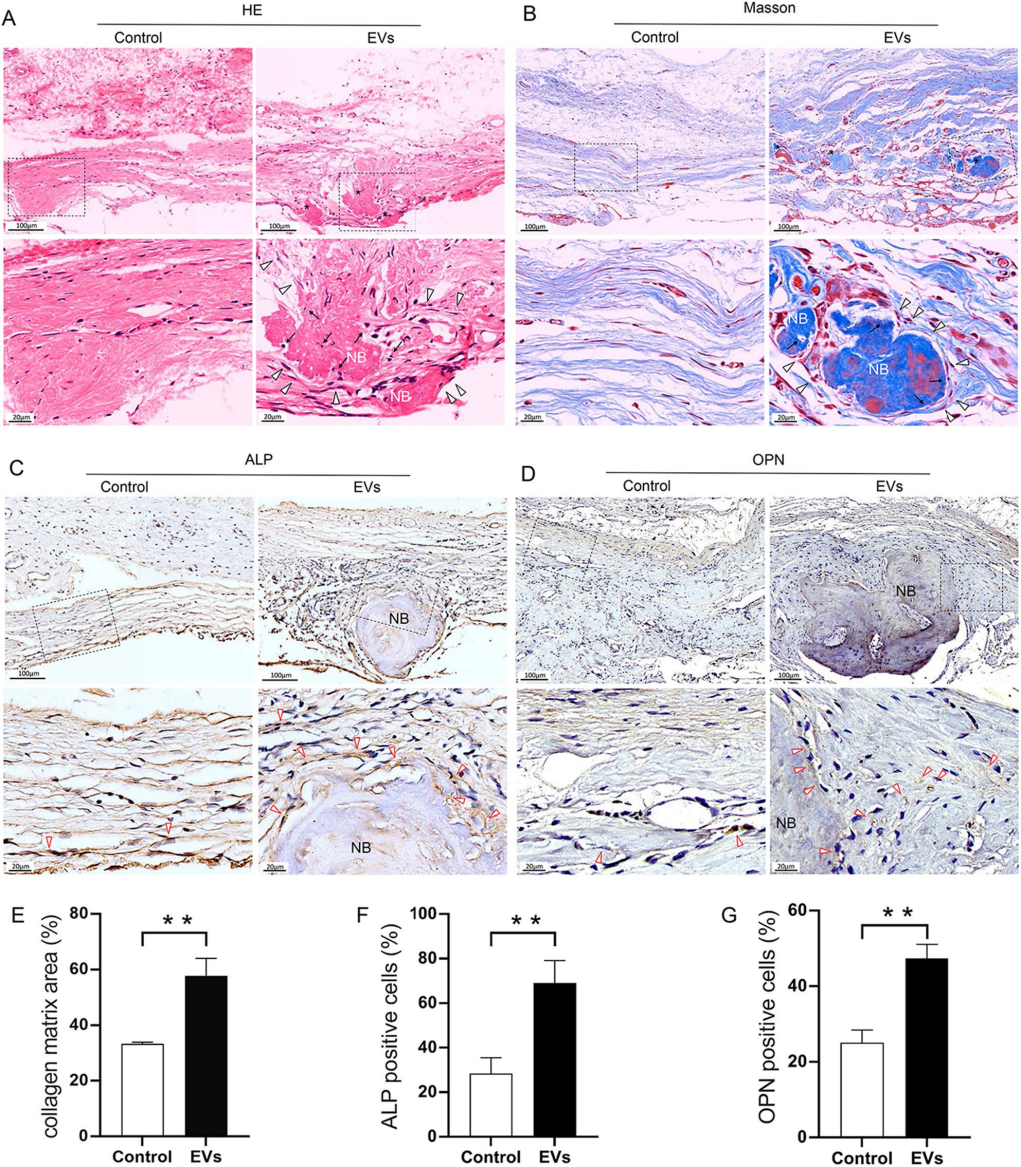
P-EVs enhanced osteogenic differentiation in rat calvaria in vivo. Representative (**A**) hematoxylin and eosin (H&E) and (**B**) Masson’s staining histological images at 8 weeks post-implantation. The bone defects were mainly filled with collagen-rich fibrotic connective tissue in the control group. New bone (NB)-like tissue (black star) formation was observed in the EV-treated group. The collagen matrix was stained blue and mature mineralized matrix was stained red. Under high magnification, osteoblast-like cells (black triangle) were observed along the mineralized matrix, and osteocyte-like cells (black arrow) were embedded in it. Immunohistochemical (IHC) staining for the osteogenic markers (**C**) ALP and (**D**) osteopontin (OPN). The brown granules marked by red triangles indicate positive staining. Semi-quantitative analysis of Masson’ staining (**E**) and IHC staining (**F**, **G**).Scale = 100 μm at low magnification and 20 μm at high magnification.

Histological evaluation of new bone formation in rat calvaria showed that P-EVs enhanced osteogenic differentiation in vivo. H&E staining at 8 weeks post implantation revealed new bone-like tissue formation in the EV-treated group, whereas the bone defect was filled with collagen-rich fibrotic connective tissue in the control group (Fig. 5A). Masson’s staining revealed that a significantly larger amount of denser collagen and mineralized matrix structures were formed in the EV group than in the control group (Fig. 5B, E). IHC staining indicated that EV implantation was associated with two key osteogenic markers, ALP and OPN (Fig. 5C, D). EV implantation group showed a significant increase in the percentage of osteogenic ALP positive (Fig. 5F) and OPN positive cells (Fig. 5G).

### Internalization of P-EVs by BMMSCs via the lipid raft/cholesterol endocytosis pathway

**Figure 6:**
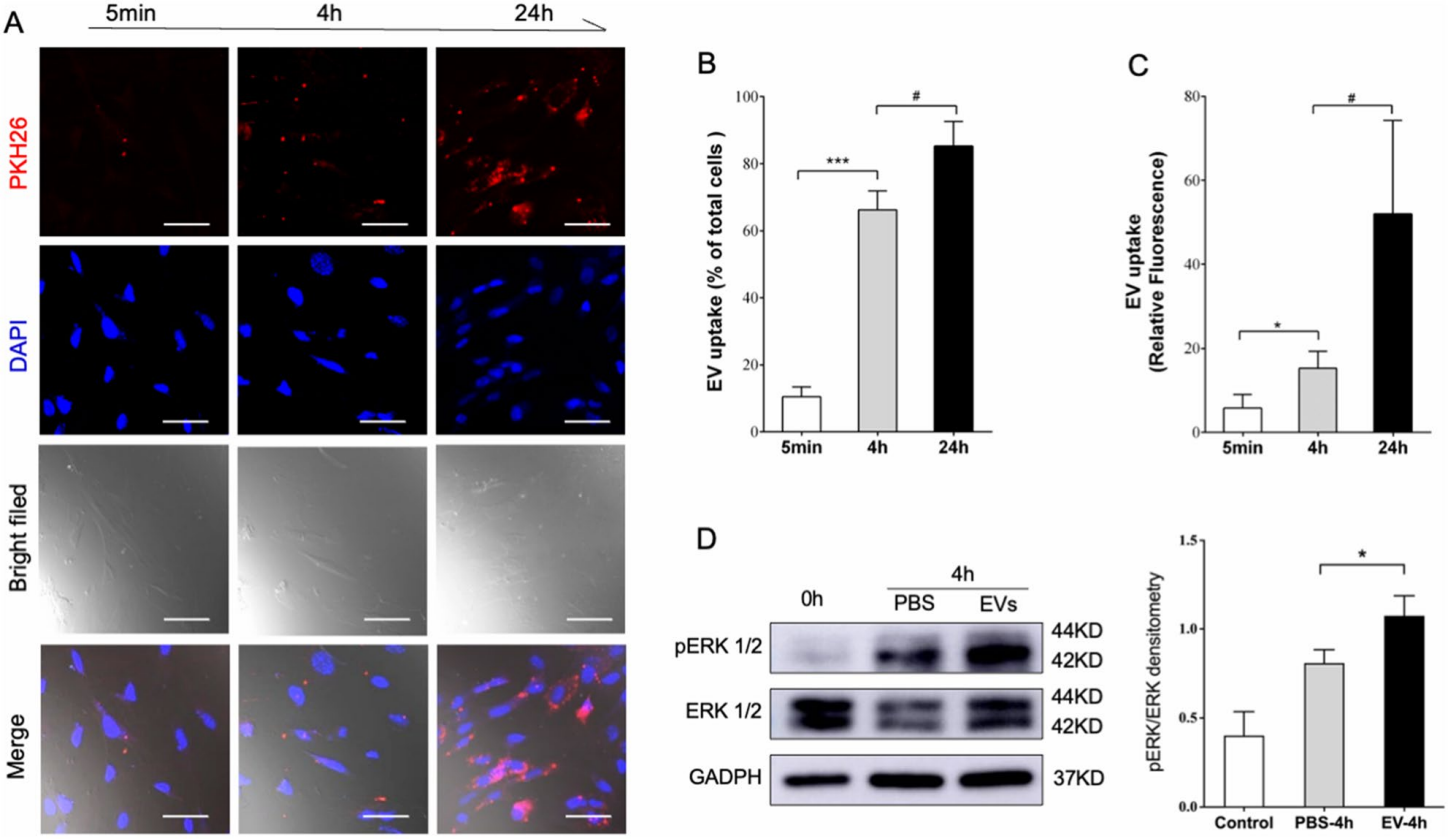
EV internalization activated extracellular signal-regulated kinase (ERK) phosphorylation in BMMSCs. (**A**) Immunofluorescence imaging showing PKH26-labeled EVs (red) in BMMSCs after 5 min, 4 h, and 24 h of incubation. Cells were stained with 4′,6-diamidino-2-phenylindole (DAPI, blue). Scale bars = 10 μm. (**B**) Percentage of cells that uptake red-labeled EVs after 24 h of EV incubation. (**C**) Relative fluorescence intensity increased after 24 h of EV treatment. Data are expressed as mean ± standard deviation of triplicate experiments. (**D**) Western blotting showing that EV treatment activates ERK1/2 phosphorylation in BMMSCs.

To observe P-EV uptake by BMMSCs, PKH-26-labeled EVs were co-incubated with BMMSCs for different periods, and internalization was observed under confocal microscopy. As shown in Fig. 6A–C, EV uptake by BMMSCs was observed as early as 5 min after incubation. Around 66.3 ± 3.2% of BMMSCs took up the fluorescence-labeled EVs after 4 h of incubation. The intracellular accumulation of EVs increased with incubation time. At 24 h post-incubation, most BMMSCs (85.2 ± 4.3%) had internalized P-EVs distributed in the perinuclear region of the cytoplasm. EV internalization activated ERK1/2 phosphorylation in BMMSCs (Fig. 6D).

**Figure 7:**
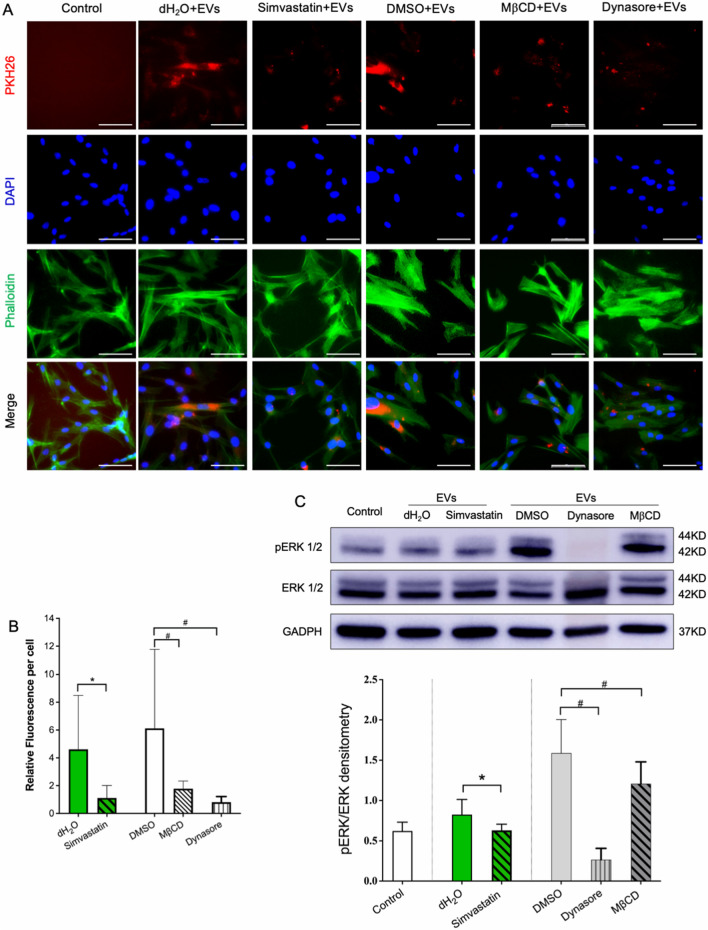
P-EVs enter BMMSCs through the cholesterol-dependent endocytosis pathway. (**A**) Fluorescence imaging depicting the influence of endocytosis inhibitor simvastatin, MβCD, and dynasore on EV uptake by BMMSCs. Control cells were incubated with dimethyl sulfoxide (DMSO) or phosphate-buffered saline (PBS). Scale bar = 50 μm. (**B**) EV internalization was significantly inhibited by simvastatin, MβCD, and dynasore. *p < 0.05, as compared with the PBS control; #*p* < 0.05, as compared with the DMSO control. (**C**) Western blotting revealed that inhibition of EV internalization attenuated ERK1/2 phosphorylation.

To explore the internalization mechanism of endocytic vesicle-mediated P-EV entry, BMMSCs were pretreated with simvastatin (an inhibitor of cholesterol synthesis), dynasore (a dynamin-dependent endocytosis inhibitor), or MβCD (a cholesterol-depleting agent). Fluorescence imaging revealed that EV uptake was significantly inhibited by dynasore and simvastatin and partially inhibited by MβCD (Fig. 7A, B). As shown in Fig. 7C, western blotting revealed that inhibition of EV internalization attenuated ERK1/2 phosphorylation (dynasore by 8.3 folds, MβCD by 1.3 folds, and simvastatin by 1.3 folds). These results indicate that BMMSCs mediate P-EV endocytosis through lipid raft/cholesterol endocytosis pathways involving dynamin and cholesterol.

## Discussion

Although MSCs were initially considered to exert therapeutic effects through cell differentiation and cell replacement by acting as seed cells, increasing evidence suggests that their effects are mainly mediated by paracrine factors. In fact, PDLSCs did not induce M1 polarization but could induce M2 polarization via paracrine products^12^. It is now apparent that MSCs exert many, if not most, paracrine effects by releasing EVs^13^. However, the secretion and uptake mechanisms of P-EVs, which are emerging as promising therapeutic agents for bone remodeling regulation, remain unclear. Therefore, this study aimed to examine the impact of P-EVs on the osteogenic differentiation of BMMSCs.

EVs are small phospholipid membrane-enclosed vesicles originating from inward germination of endosomal membranes. Following the fusion of intracellular multivesicular bodies with the plasma membrane, these vesicles are secreted into the extracellular space where they serve as messengers of intercellular communication^23^. In our study, EV biogenesis from PDLSCs was observed using TEM. SEM provided three-dimensional surface topology information of the vesicles on the surface of PDLSCs, which further evidenced that these were EVs released by PDLSCs into the extracellular space.

The parent cell type of the EVs plays an important role in bone biology. MSC-derived EVs contribute to fracture healing, whereas osteosarcoma-derived EVs do not^24^. PDLSCs are good candidates for bone regeneration for both calvarial and alveolar bone defects^5,25^. However, PDLSCs indirectly participate in periodontal bone regeneration^6,7^. MSC paracrine effects are considered a promising novel regenerative therapy for periodontal regeneration^26^. In fact, transplantation of PDLSC-derived conditioned medium containing EVs and other paracrine factors can induce periodontal bone regeneration^11^. To identify the effects of PDLSC paracrine factors on osteogenesis, an in vitro co-culture system was developed. PDLSCs were cultured in the upper insert, whose EVs could pass through the Transwell pore, with BMMSCs acting as the host tissue. In this context, gene expression changes in and mineralization of BMMSCs may reflect the influence of PDLSC paracrine signaling. These findings indicate that the paracrine effects of implanted PDLSCs have a significant impact on the activity of local host cells and at least govern regeneration in this manner^7,12^.

EV formation and secretion are complex biological processes. Rab27a is required for the fusion of multivesicular bodies with the plasma membrane to release EVs from cells^27^.

It was the first identified Rab protein whose dysfunction leads to type 2 Griscelli syndrome, a human hereditary disease^28^. A previous study reported that Rab27a silencing inhibited sEV secretion without modifying sEV protein composition^22^. To evaluate the role of EV secretion in the upregulation of BMMSC osteogenesis by co-culture, siRNA was used to knockdown Rab27a, a member of the small GTPase Rab family and a promoter of EV secretion^22,28^. By inhibiting EV release, PDLSC^*siRab27a*^ significantly eliminated the co-culture-upregulated osteogenesis of BMMSCs, which is consistent with a previous study showing that Rab27a knockdown decreased the production of EVs and immunosuppression of dendritic cell function^29^.

As co-culturing with PDLSCs enhanced BMMSC osteogensis via Rab27a -mediated EV secretion, EVs were isolated from PDLSCs and their influence on BMMSCs was examined. Both ultracentrifugation and commercial isolation kits, such as Exo-Quick, can be used to isolate EVs^30^. Combination differential ultracentrifugation with microfiltration could effectively isolate the vesicles from contaminating proteins in the cell culture medium^16^. The present data indicate that isolated EVs mainly comprised sEVs. However, because of the overlapping properties of sEVs and other EVs, and our inability to ascertain that the purified vesicles were only sEVs, we refer to them as EVs throughout this article^31–33^. In EV-based intercellular communication, the recipient cell type is an important factor^22^. Considering the function of the PDL-bone interface in the periodontium, BMMSCs were the focus of the present study. P-EVs enhanced BMMSC differentiation and bone fracture healing as demonstrated in both in vivo and in vitro experiments. Consistent with previous research, EVs derived from mesenchymal progenitor cells were shown to induce bone healing by enhancing resident cellular functions or vasculogenic responses^24,26^. PDLSCs participate in periodontal bone remodeling through EV release, which is associated with the accommodation of bones to dynamic mechanics. It is likely that in the periodontal microenvironment, P-EVs contribute to bone remodeling under physiological and pathological conditions.

EV entry into recipient cells was verified using confocal microscopy. Endocytosis is a physiological process of internalization of molecules and surface proteins mediated by endocytic vesicles^34^. Similar to the results of previous studies, the process of EV entry into cells is time-dependent, and the endocytic process can reach saturation^35,36^. A recent study showed that P-EVs could be taken up by macrophages after 1 h of treatment^19^. In the present study, P-EV visualization within BMMSCs 5 min post-administration suggested extremely rapid internalization, which increased up to 24 h. The different kinetics of EV uptake might be related to recipient cell types and different processes^20,23^. The mechanisms of EV internalization include macropinocytosis, phagocytosis, clathrin-mediated endocytosis, vacuole-mediated endocytosis, and lipid raft-mediated endocytosis ^20^. The cargo and function of EVs are highly dependent on the producing parent cells. Internalization of glioblastoma cell-derived EVs induces the phosphorylation of ERK1/2, a downstream target associated with lipid rafts^37^. P-EVs were also reported to upregulate ERK1/2 phosphorylation in recipient cells such as macrophages and BMMSCs^19,21^. Upregulation of ERK1/2 phosphorylation, a predictive pathway of mitogen-activated protein kinase, is regularly linked to MSC osteogenesis^38^. In this study, the activation of ERK1/2 phosphorylation was regarded as an indicator of EV endocytosis. The role of lipid raft endocytosis in P-EV uptake by BMMSCs was examined using chemical inhibitors. Lipid raft endocytosis is a dynamically dependent process mediated by cholesterol-rich domains^39^. As a key component of lipid rafts, cholesterol is essential for the pharmacological inhibition of lipid raft-mediated endocytosis. This includes the use of simvastatin to inhibit cholesterol synthesis and MβCD to extract cholesterol from the plasma membrane. Raft-dependent endocytic pathways can be classified according to their caveolin- and dynamin-dependence^40^. P-EV internalization was inhibited by dynasore, suggesting that the process is dynamin-dependent.

## Conclusions

**Figure 8:**
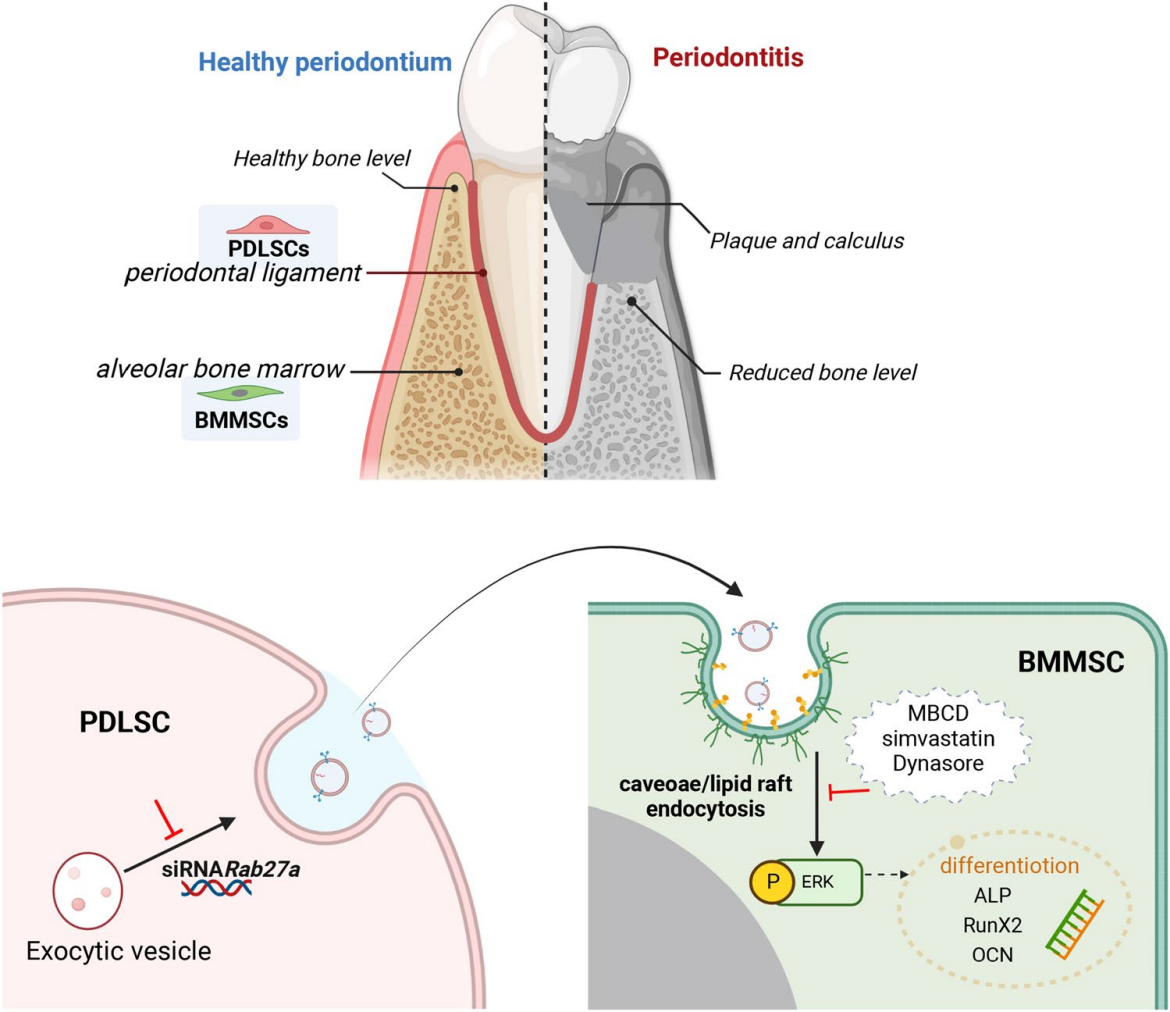
Schematic diagram of the cellular communication between PDLSCs and BMMSCs in periodontium. PDLSCs contribute to BMMSC osteogenesis via Rab27a-mediated EV secretion. EVs are internalized by BMMSCs, activating ERK1/2 phosphorylation, mainly through caveolae/lipid raft endocytosis.

In conclusion, our findings demonstrate for the first time that PDLSCs secrete EVs via Rab27a, which participates in the osteogenesis of BMMSCs and EV uptake by BMMSCs in a caveolae/lipid raft-dependent manner (Fig. 8). These findings provide new perspectives on the cellular communication of PDLSCs and BMMSCs as well as new clues for the potential use of EVs as therapeutic interventions for bone regeneration in the periodontium microenvironment. However, some limitations of this study still need to be addressed. First, unlike immortalized cell lines, PDLSCs are primary cells with individual differences and a limited lifespan^31^. Their cell state, including cell passage and donor age, should be considered. Second, EV cargo is highly complex and diverse in different source cells. Rab27aKD might change the cargo of P-EVs, such as miRNA and proteins. Specific factors, such as protein and RNA, are thought to be identified in P-EVs. Future studies should consider source cell information, the optimum method for EV isolation, and the measurement of bioactive molecules.

## Supplementary Information

Supplementary Information 1.

Supplementary Information 2.

## Data Availability

All data generated or analyzed during this study are included in this published article and its supplementary materials.

## References

[1] Ho SP (2010). The biomechanical characteristics of the bone-periodontal ligament-cementum complex. Biomaterials.

[2] Ho SP, Marshall SJ, Ryder MI, Marshall GW (2007). The tooth attachment mechanism defined by structure, chemical composition and mechanical properties of collagen fibers in the periodontium. Biomaterials.

[3] Bosshardt DD (2005). Are Cementoblasts a subpopulation of osteoblasts or a unique phenotype?. J. Dent. Res..

[4] Trubiani O (2016). Alternative source of stem cells derived from human periodontal ligament: A new treatment for experimental autoimmune encephalomyelitis. Stem Cell Res. Ther..

[5] Seo BM (2004). Investigation of multipotent postnatal stem cells from human periodontal ligament. Lancet.

[6] Yu N (2013). Enhanced periodontal tissue regeneration by periodontal cell implantation. J. Clin. Periodontol..

[7] Yu N (2015). Periodontal cell implantation contributes to the regeneration of the periodontium in an indirect way. Tissue Eng. Part A.

[8] Zhang H (2016). Composite cell sheet for periodontal regeneration: Crosstalk between different types of MSCs in cell sheet facilitates complex periodontal-like tissue regeneration. Stem Cell Res. Ther..

[9] Aghaloo TL (2010). Osteogenic potential of mandibular vs. long-bone marrow stromal cells. J. Dent. Res..

[10] Matsubara T (2005). Alveolar bone marrow as a cell source for regenerative medicine: Differences between alveolar and iliac bone marrow stromal cells. J. Bone Miner. Res..

[11] Nagata M (2017). Conditioned medium from periodontal ligament stem cells enhances periodontal regeneration. Tissue Eng. Part A.

[12] Liu J (2022). Periodontal ligament stem cells promote polarization of M2 macrophages. J. Leukoc. Biol..

[13] Lener T (2015). Applying extracellular vesicles based therapeutics in clinical trials–an ISEV position paper. J. Extracell. Vesicles.

[14] Wang C (2022). Effects of extracellular vesicles from osteogenic differentiated human BMSCs on osteogenic and adipogenic differentiation capacity of naïve human BMSCs. Cells.

[15] Ko K (2021). Integrated bioactive scaffold with polydeoxyribonucleotide and stem-cell-derived extracellular vesicles for kidney regeneration. ACS Nano.

[16] Hettich BF, Ben-Yehuda GM, Werner S, Leroux JC (2020). Exosomes for wound healing: Purification optimization and identification of bioactive components. Adv. Sci..

[17] Wang Z (2019). Cyclic stretch force induces periodontal ligament cells to secrete exosomes that suppress IL-1β production through the inhibition of the NF-κB signaling pathway in macrophages. Front. Immunol..

[18] Xu XY (2020). Exosomes derived from P2X7 receptor gene-modified cells rescue inflammation-compromised periodontal ligament stem cells from dysfunction. Stem Cells Transl. Med..

[19] Huang H (2022). Mechanical force-promoted osteoclastic differentiation via periodontal ligament stem cell exosomal protein ANXA3. Stem Cell Rep..

[20] Ginini L, Billan S, Fridman E, Gil Z (2022). Insight into extracellular vesicle-cell communication: From cell recognition to intracellular fate. Cells.

[21] Zhao B (2022). Periodontal ligament stem cell-derived small extracellular vesicles embedded in matrigel enhance bone repair through the adenosine receptor signaling pathway. Int. J. Nanomed..

[22] Ostrowski M (2010). Rab27a and Rab27b control different steps of the exosome secretion pathway. Nat. Cell Biol..

[23] Hazan-Halevy I (2015). Cell-specific uptake of mantle cell lymphoma-derived exosomes by malignant and non-malignant B-lymphocytes. Cancer Lett..

[24] Furuta T (2016). Mesenchymal stem cell-derived exosomes promote fracture healing in a mouse model. Stem Cells Transl. Med..

[25] Rakian A, Rakian R, Shay AE, Serhan CN, Van Dyke TE (2022). Periodontal stem cells synthesize maresin conjugate in tissue regeneration 3. J. Dent. Res..

[26] Chew J (2019). Mesenchymal stem cell exosomes enhance periodontal ligament cell functions and promote periodontal regeneration. Acta Biomater..

[27] Hao M (2021). Autophagy blockade limits HER2+ breast cancer tumorigenesis by perturbing HER2 trafficking and promoting release via small extracellular vesicles. Dev. Cell.

[28] Zhang L, Zhang X, Hsieh LS, Lin TV, Bordey A (2021). Rab27a-dependent paracrine communication controls dendritic spine formation and sensory responses in the barrel cortex. Cells.

[29] Salimu J (2017). Dominant immunosuppression of dendritic cell function by prostate-cancer-derived exosomes. J. Extracell. Vesicles.

[30] Caponnetto F (2016). Size-dependent cellular uptake of exosomes. Nanomedicine.

[31] Witwer KW (2019). Defining mesenchymal stromal cell (MSC)-derived small extracellular vesicles for therapeutic applications. J. Extracell. Vesicles.

[32] Théry C (2018). Minimal information for studies of extracellular vesicles 2018 (MISEV2018): A position statement of the international society for extracellular vesicles and update of the MISEV2014 guidelines. J. Extracell. Vesicles.

[33] Xu J (2019). Human perivascular stem cell-derived extracellular vesicles mediate bone repair. Elife.

[34] Ou Q (2021). TcpC inhibits neutrophil extracellular trap formation by enhancing ubiquitination mediated degradation of peptidylarginine deiminase 4. Nat. Commun..

[35] Huang C, Narayanan R, Alapati S, Ravindran S (2016). Exosomes as biomimetic tools for stem cell differentiation: Applications in dental pulp tissue regeneration. Biomaterials.

[36] Huang C (2020). Evaluating the endocytosis and lineage-specification properties of mesenchymal stem cell derived extracellular vesicles for targeted therapeutic applications. Front. Pharmacol..

[37] Svensson KJ (2013). Exosome uptake depends on ERK1/2-heat shock protein 27 signaling and lipid raft-mediated endocytosis negatively regulated by caveolin-1. J. Biol. Chem..

[38] Hodgkinson T (2021). The use of nanovibration to discover specific and potent bioactive metabolites that stimulate osteogenic differentiation in mesenchymal stem cells. Sci. Adv..

[39] Kumari S, Mg S, Mayor S (2010). Endocytosis unplugged: Multiple ways to enter the cell. Cell Res..

[40] French KC, Antonyak MA, Cerione RA (2017). Extracellular vesicle docking at the cellular port: Extracellular vesicle binding and uptake. Semin. Cell Dev. Biol..

