# Supplementary material for: Rab27a-mediated extracellular vesicle secretion contributes to osteogenesis in periodontal ligament-bone niche communication
Source: Sci Rep. 2023 May 25;13:8479. doi: 10.1038/s41598-023-35172-x (PMC10213006; doi:10.1038/s41598-023-35172-x)
Supplement: Supplementary file 1 — Supplementary Information 1. [file 41598_2023_35172_MOESM1_ESM.docx]

**Supplementary information**

**
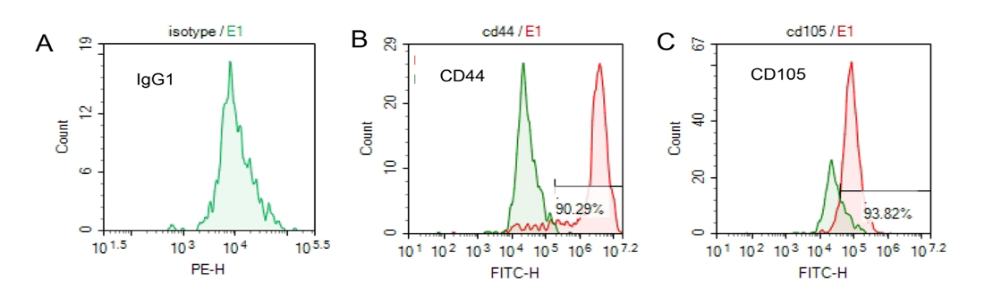
**

**Supplementary Figure 1, related to Figure 1**

Using FITC IgG1 Isotype (A) as control, PDLSCs positively expressed CD44 (B) and CD105 (C). The strong positive markers of MSCs, CD44 and CD105, was analyzed by flow cytometry to complement CD73, CD90 and CD146 for more rigorous identification of PDLSCs. Antibodies: FITC IgG1 Isotype (#400107, Biolegend), FITC hCD44 (#397518, Biolegend) and FITC hCD105 (#323204, Biolegend).


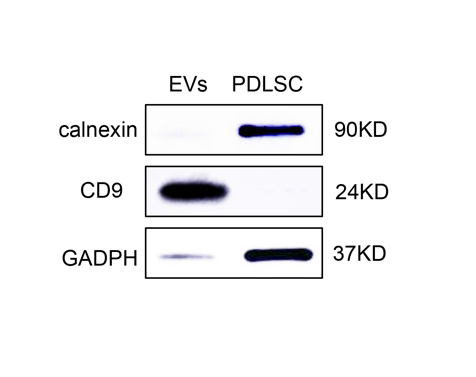


**Supplementary Figure 2, related to Figure 4**

Western blot analysis showed that P-EVs were negative for calnexin, an endoplasmic reticulum resident protein and generally absent from EVs. The primary antibody used were anti-calnexin (1:200, #23954, Santa cruz). According to ISEV 2018 (reference 32), 6 markers were evaluated to demonstrate the presence of EVs: category 1a (CD63, CD8), category 1b (CD9), category 2a (TSG101), category 2b (GADPH) and category 4c (calnexin).
