# Supplementary figures and images for: Rab27a-mediated extracellular vesicle secretion contributes to osteogenesis in periodontal ligament-bone niche communication
Source: Sci Rep. 2023 May 25;13:8479. doi: 10.1038/s41598-023-35172-x (PMC10213006; doi:10.1038/s41598-023-35172-x)

Figure 2B

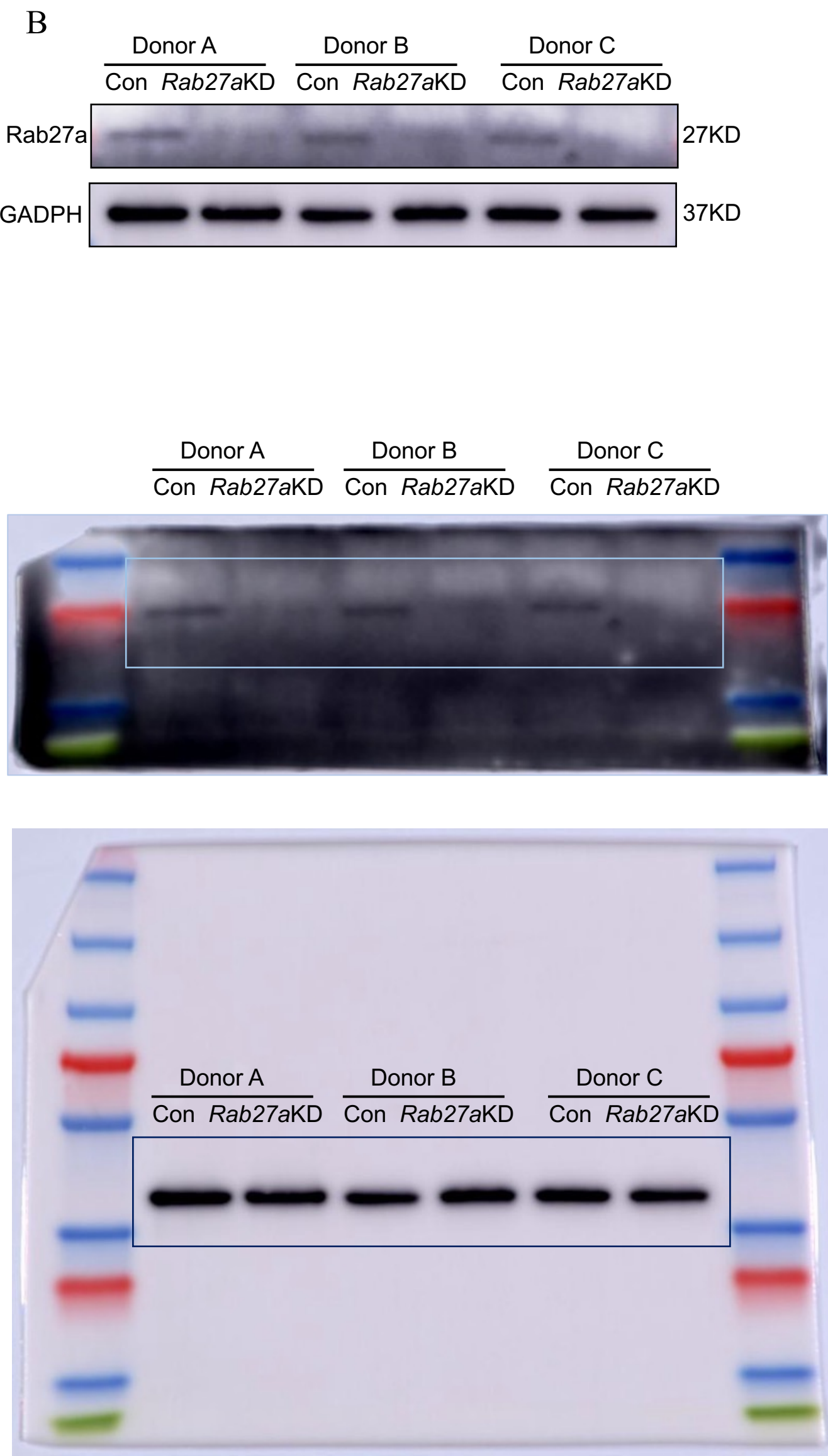

Figure 3D

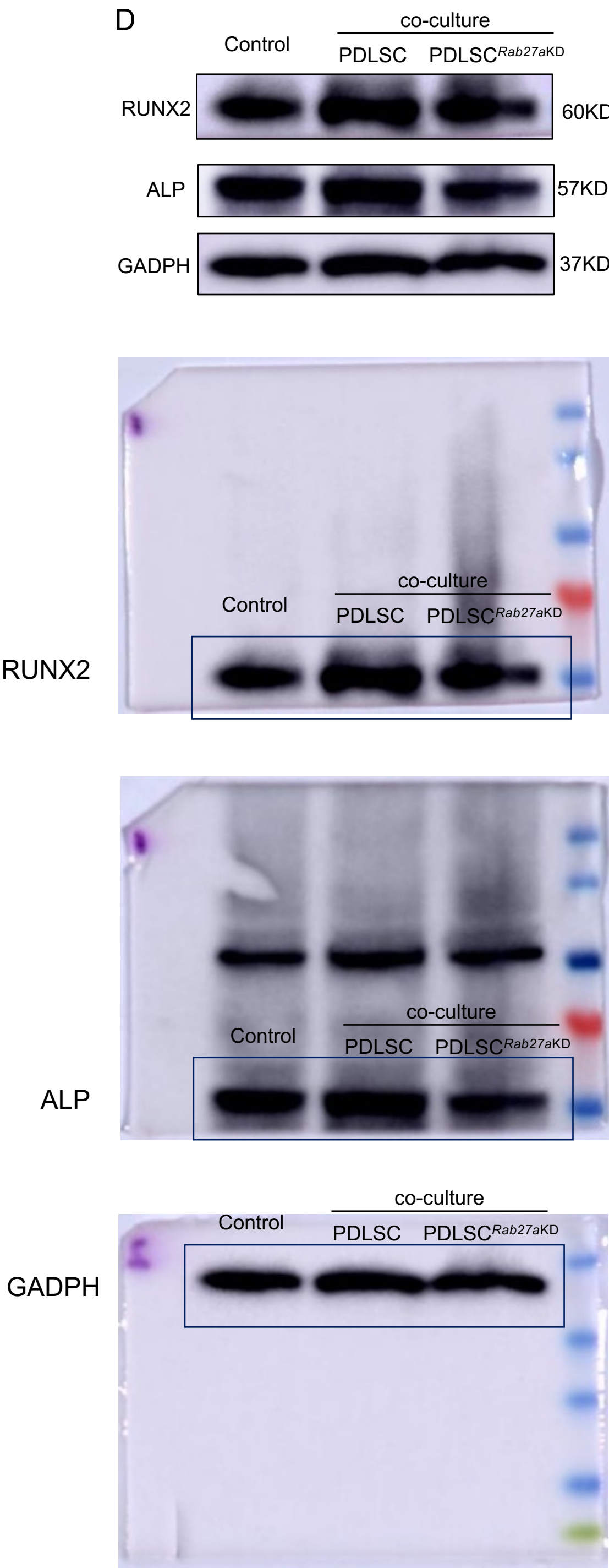

Figure 4C

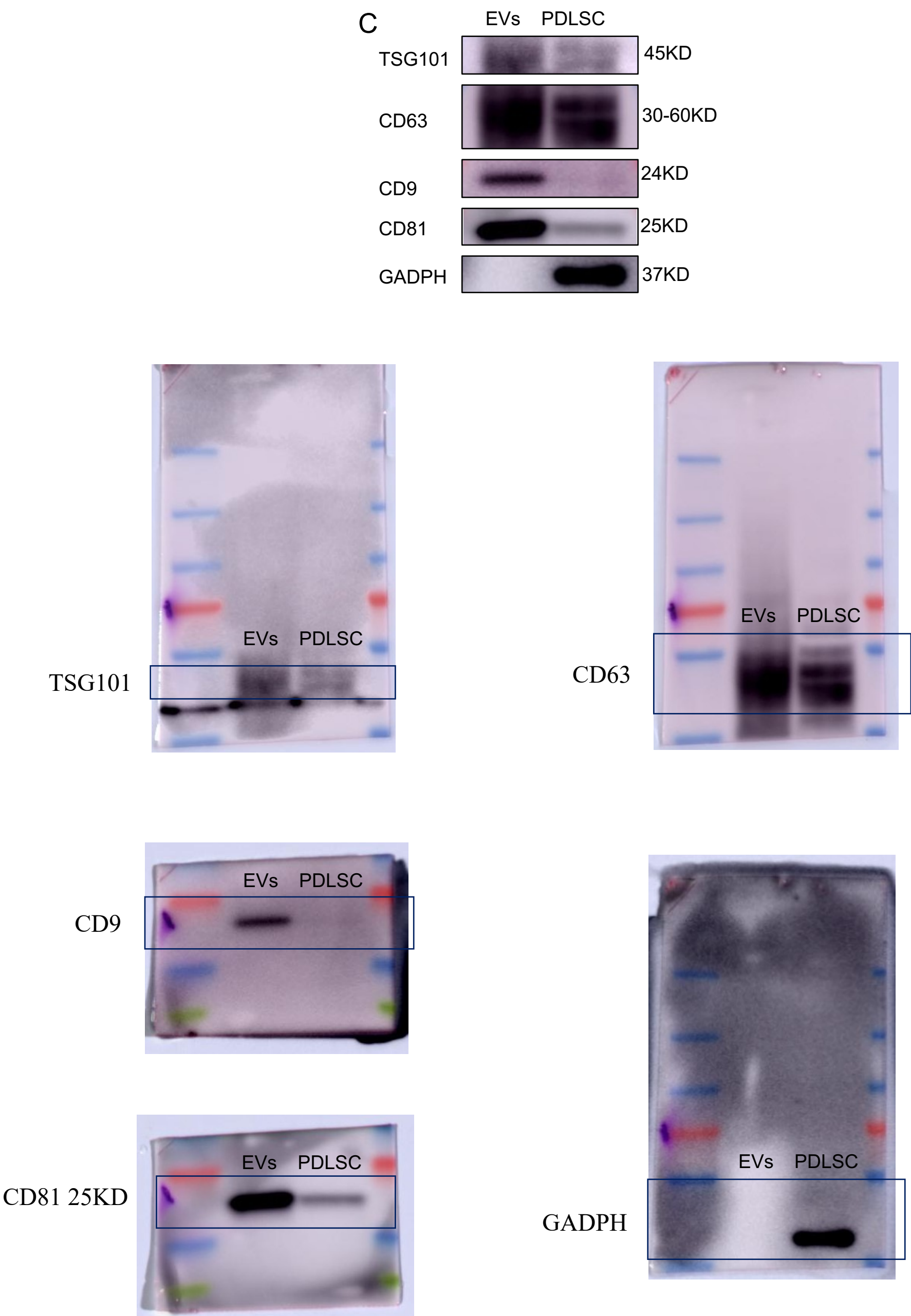

Figure 6D

D

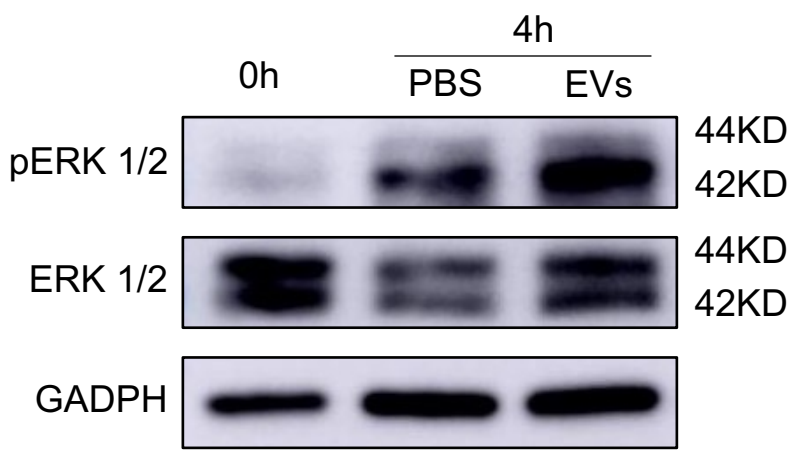

pERK 1/2

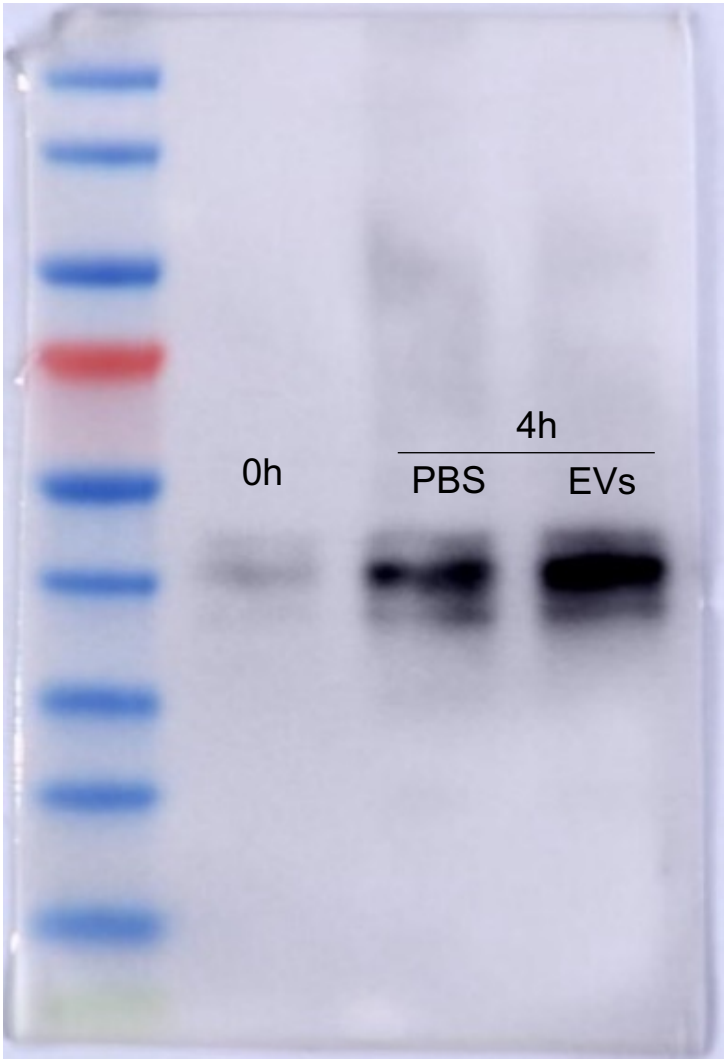

ERK 1/2

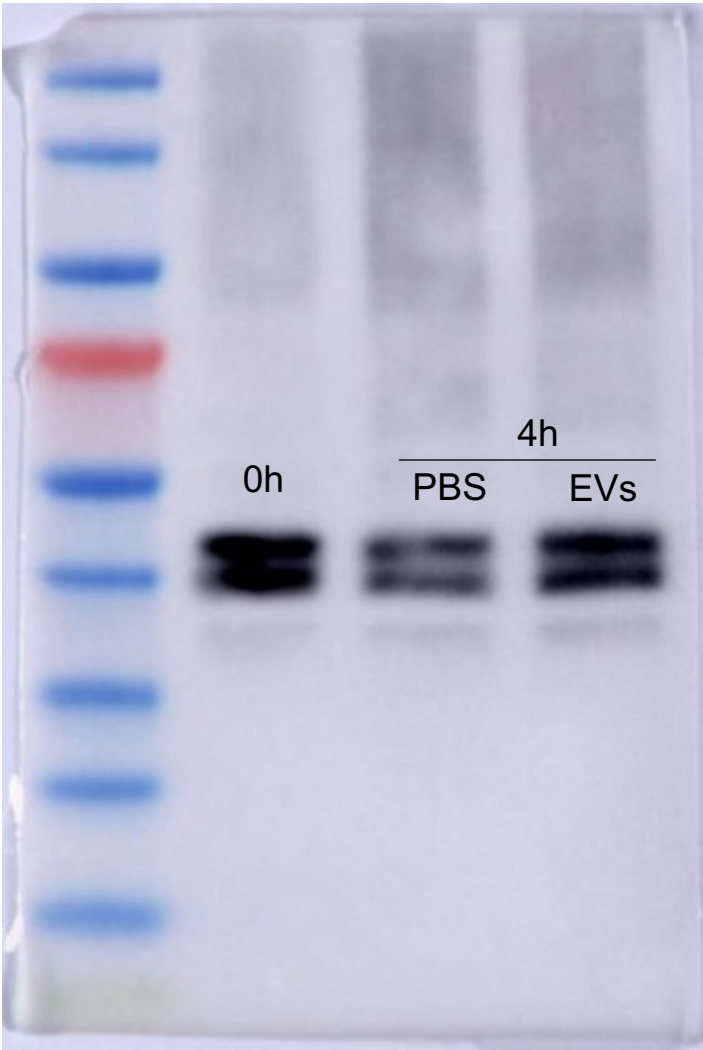

GADPH

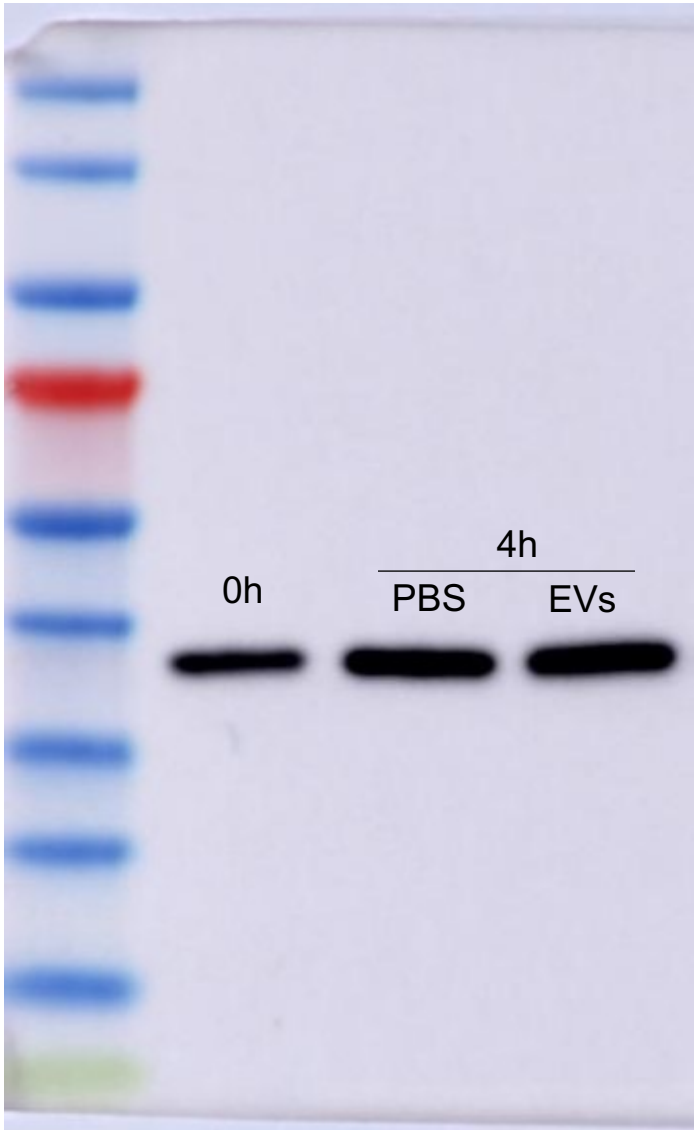

Figure 7C

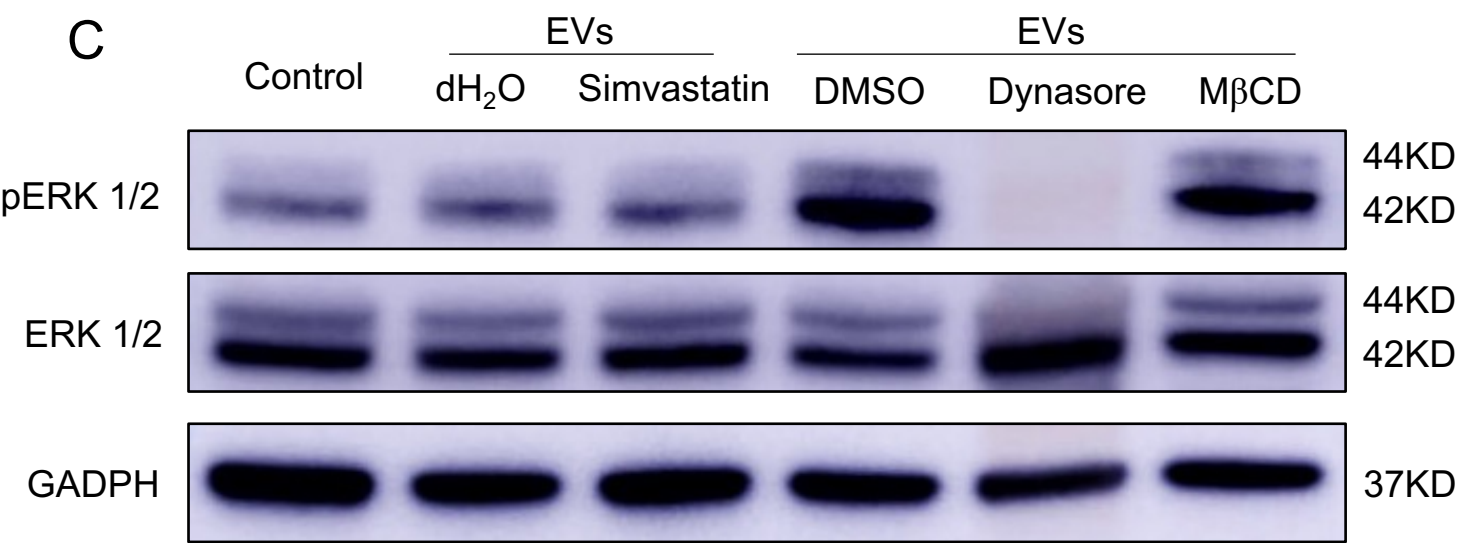

pERK 1/2

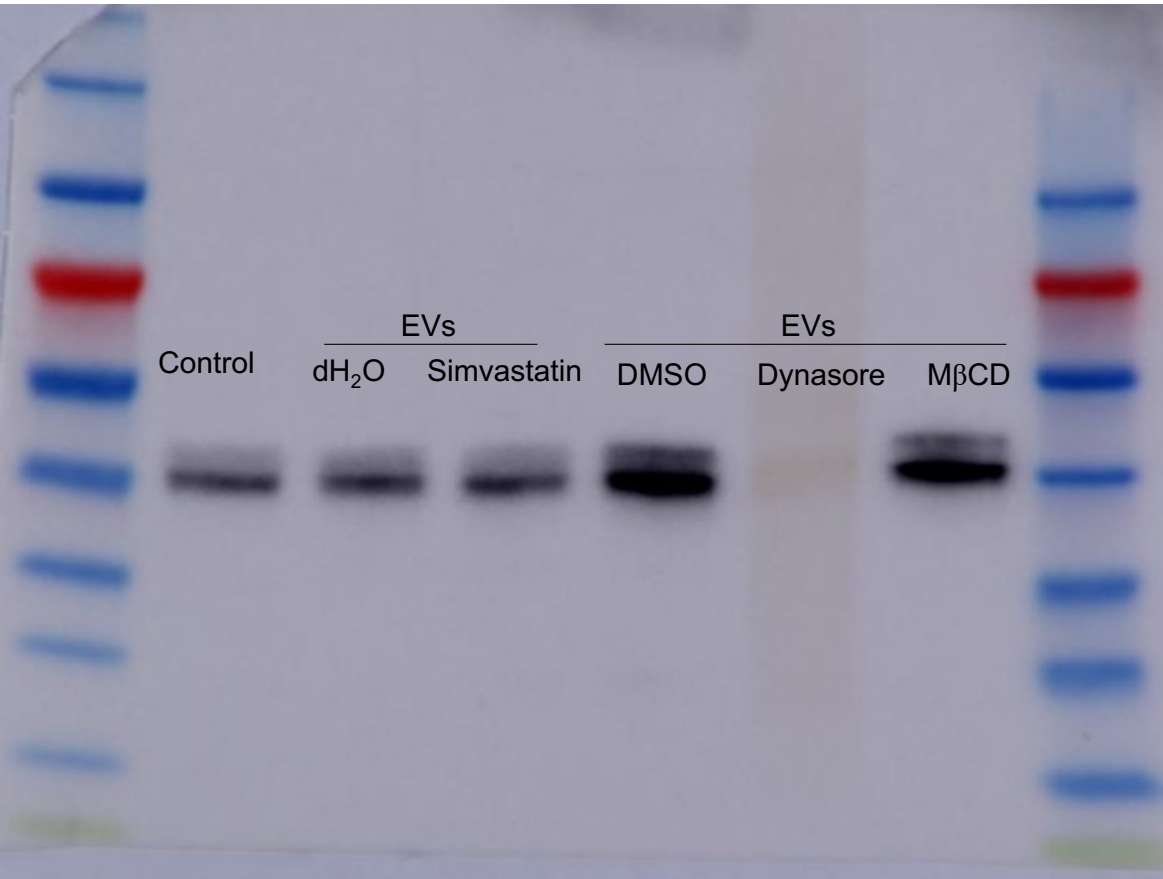

ERK 1/2

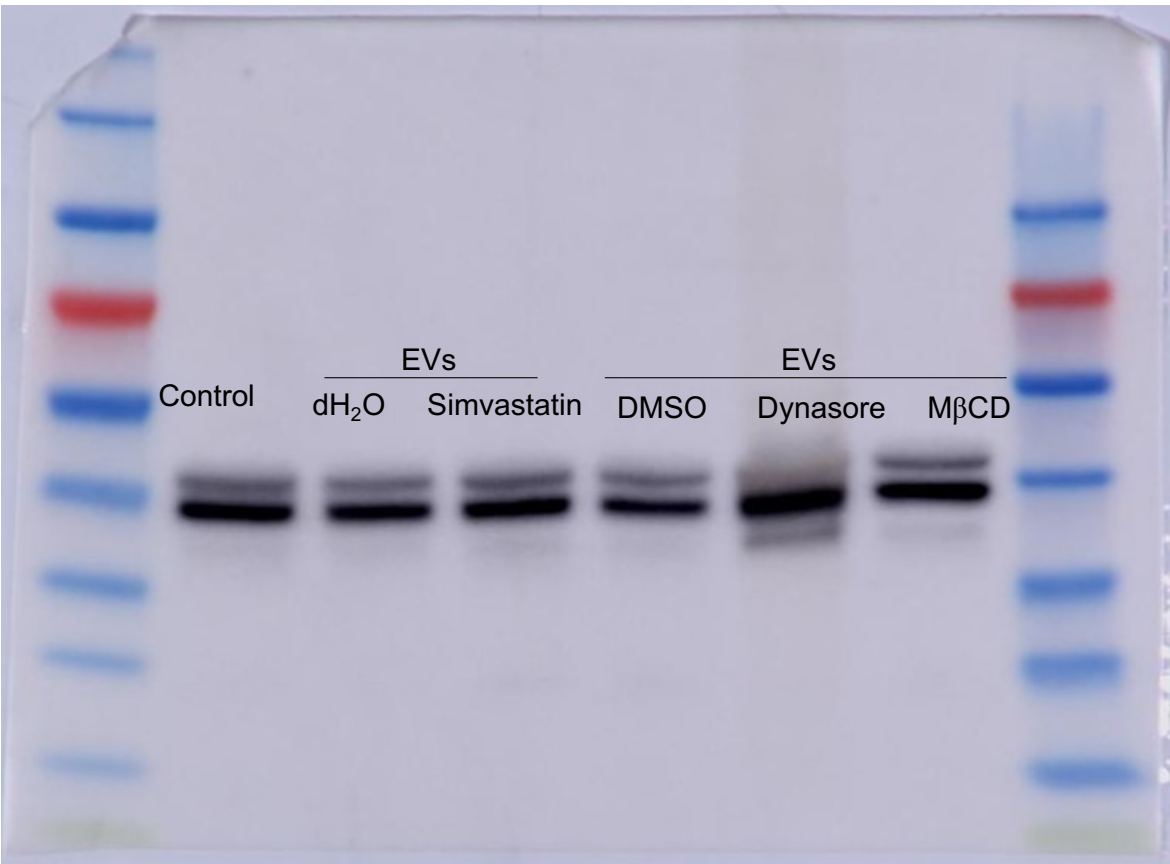

GADPH

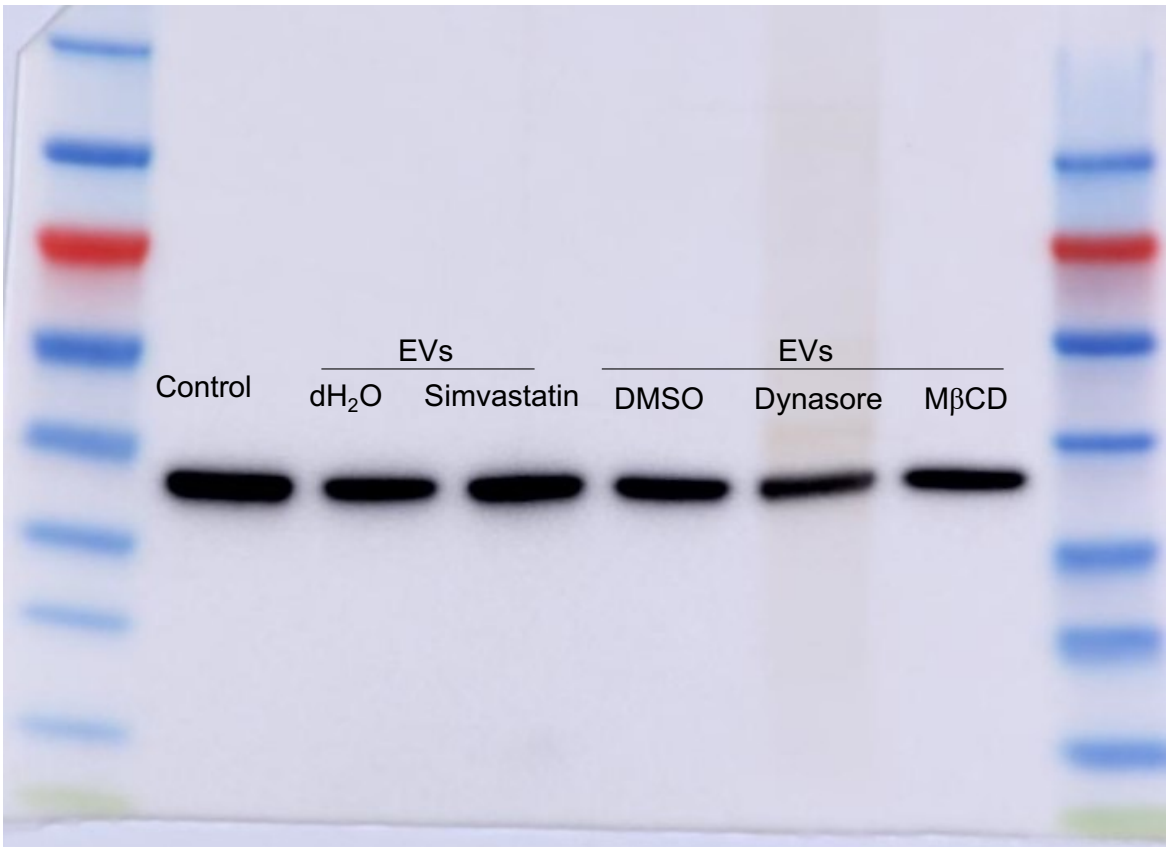

Supplement: Supplementary file 2 — Supplementary Information 2. [file 41598_2023_35172_MOESM2_ESM.pdf]
